# The Kinetic Consequences of Water on Catalytic Methane Pyrolysis

**DOI:** 10.1021/acscatal.6c00011

**Published:** 2026-02-26

**Authors:** Phuong T. Nguyen, Caleb Q. Bavlnka, Laura A. Gomez, Thy L. T. Ho, Ismaeel Alalq, Bin Wang, Daniel Resasco, Steven P. Crossley

**Affiliations:** School of Sustainable Chemical, Biological and Material Engineering, 6187 University of Oklahoma, Norman, Oklahoma 73019, United States

**Keywords:** hydrogen production, carbon nanotubes, catalytic methane decomposition, water cofeeding, nickel-based catalysts

## Abstract

Hydrogen production from biomass and natural gas has emerged as a prominent research area in response to the growing demand for energy from alternative sources that minimize CO_2_ emissions. In this study, we investigate the impact of water, which is present in and generated from biomass-derived streams, on carbon nanotube (CNT) growth and hydrogen production during methane decomposition using Ni–Mo/MgO as a catalyst. We reveal here that the role of water on CNT growth is highly complex; its effect depends on the stage of growth at which the water is incorporated. When water is introduced at the beginning of methane decomposition (*t* = 0 h), methane conversion rates are negatively impacted. We hypothesize that water inhibits the significant phase changes the Ni–Mo/MgO catalyst undergoes during catalyst carburization. In contrast, the incorporation of a small percentage of water after a stabilization period (*t* = 3 h) results in methane conversion rate enhancements that scale with the introduced water partial pressure as water selectively reacts with amorphous carbon deposits that lead to catalyst deactivation, thus prolonging the lifetime of some of the most active sites. Moreover, water incorporation after stabilization significantly reduces the apparent activation energy. Density Functional Theory (DFT) calculations reveal that water preferentially interacts with carbon fragments on the catalyst surface to remove carbon deposits with a barrier lower than that required for methane activation, further supporting its role in cleaning active sites on the catalyst surface. Characterization of the resulting carbon nanotubes reveals the formation of more graphitic materials produced in the presence of water, highlighting the impact of water on nanotube properties. These results provide clarity toward the many ways in which water, or cofeeding of biomass-derived materials, may impact catalytic methane pyrolysis rates.

## Graphical abstract

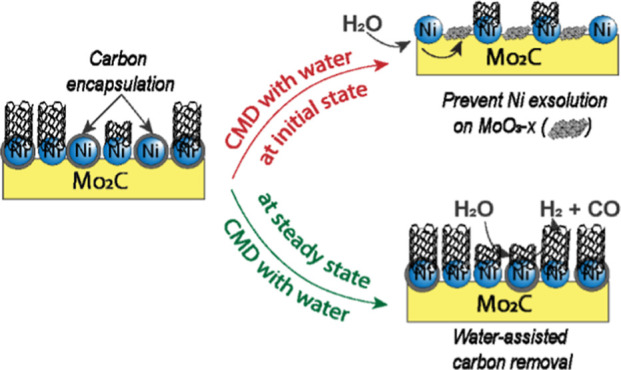

## 1 Introduction

Clean hydrogen is projected to account for as much as 12% of global energy consumption by 2050.^1,2^ Currently, most industrial hydrogen is produced through steam methane reforming (SMR), a process in which methane reacts with steam to produce hydrogen. However, this process generates substantial CO_2_ emissions, significantly contributing to the rise of greenhouse gases and creating the need for carbon capture and storage technologies to reduce their environmental impact.^3^ Thus, significant environmental impacts highlight the need for more sustainable processes to satisfy the growing global demand for hydrogen.

Catalytic methane decomposition (CMD) offers a promising approach for hydrogen production by converting methane into CO_2_-free hydrogen and solid carbon.^3–8^ In comparison to conventional hydrogen production methods, such as SMR, CMD is more favorable thermodynamically, with an endothermic enthalpy requirement of 37.7 kJ/mol of H_2_ produced compared to the 63.4 kJ/mol of H_2_ required for SMR.^6,9^ Additionally, CMD produces valuable solid carbon byproducts in the form of carbon nanotubes (CNTs), fibers, or filaments, further enhancing its potential economic viability.^5,6,10,11^

Key challenges that limit CMD is catalyst stability and reusability. Effective catalyst reuse necessitates catalyst particles be well anchored to the support throughout nanotube growth, a process known as the base-growth mechanism.^9^ Ensuring a base-growth mechanism is essential for any follow-on continuous recovery and separation of CNTs from the catalyst. In a previous study on the dynamic interface evolution during CMD over Ni–Mo/MgO, we reported the detailed characterization of the Ni–Mo/MgO catalyst, highlighting its superior performance for CNT growth via a base-growth mechanism.^12^ We demonstrated that the formation of molybdenum carbide (Mo_2_C) during the initial stages of methane introduction plays a crucial role in facilitating a base-growth CNT synthesis mechanism as the carburization process stabilizes C-saturated Ni nanoparticles within the carbide matrix. Initial attempts at in situ separation of CNTs from various catalysts have been made, further highlighting the importance of catalyst reusability.^13,14^

Despite extensive advancements in this field, catalyst deactivation remains a significant challenge when using metal-based catalysts for methane decomposition. Two primary mechanisms contributing to reduced catalyst lifetime are metal sintering and particle encapsulation.^15–17^ Metal sintering is more prominent at elevated reaction temperatures.^18,19^ Meanwhile, metal encapsulation occurs when the rate of carbon deposition from methane onto the metal surface exceeds the rate of carbon diffusion from the point of activation to the nanotube-metal interface. This rate imbalance leads to the formation of amorphous carbon deposits that can render active sites inaccessible.^20–22^ These amorphous carbon deposits can be removed through steam gasification or combustion with oxygenated compounds to produce CO and CO_2_ while extending the catalyst lifetime.^20,23^ Thus, cofeeding oxygen-containing biomass-derived compounds could be a promising strategy to reduce catalyst deactivation during CMD while providing an opportunity for additional carbon sequestration.^24,25^ The potential to enhance rates of hydrogen production while sequestering carbon into useful materials could help in achieving the ambitious price target of $1/kg for hydrogen by 2050.^26,27^ However, we must first understand the fundamental role of molecules such as water that have been proposed to modify and extend catalyst lifetimes. Building on the insights gained from the previous study of the Ni–Mo/MgO catalyst,^12^ this work examines the impact of water on both hydrogen production and CNT formation.

While water is often introduced in order to modify CNT properties or promote ultralong nanotube growth during chemical vapor deposition,^28–31^ water is most typically cofed along with the carbon-containing feedstock, and its role in promoting intrinsic growth kinetics is typically convoluted with the critical initial stages of catalyst particle and nanotube nucleation. This leads to widely ranging and sometimes contradictory claims regarding the fundamental role of water; optimal conditions for water incorporation in enhancing growth vary as well. For example, some have claimed that the primary explanation for water-induced enhancement in growth is due to amorphous carbon removal,^21,25^ while others hypothesize that the role of water is primarily to inhibit sintering of catalyst particles.^26^ While several authors have observed increased rates of CNT generation upon water incorporation, varied results have been reported regarding the net benefits and the necessary partial pressures required to achieve CNT growth enhancements. Li et al. found that introducing water dramatically increases the defect density of carbon while decreasing total carbon yield from methane over a Fe_2_O_3_/Al_2_O_3_ aerogel catalyst. Although water was described as necessary for producing single-walled CNTs, its concentration was not explicitly stated.^32^ In contrast, Futaba et al. identified an optimal water-to-ethylene ratio of approximately 1:1000 under various reaction conditions (all ≤180 ppm of H_2_O) over a Fe/Al_2_O_3_ catalyst. They observed that higher water partial pressures led to shorter CNTs, indicating reduced catalyst lifetimes and fewer turnovers.^33^ Similarly, Wang et al. reported that high water concentrations (4670 ppm) completely prevent CNT growth while lower concentrations (from 0 to 1970 ppm) enhance methane conversion, while, contrary to other reports, decreasing CNT defect density.^30^ These studies highlight the complex role of water in CNT synthesis, as water has been reported to impact carbon yield, defect density, and catalyst stability in opposing manners across a broad range of partial pressures. This is evidence that further investigation is necessary.

Our findings indicate that whether water is beneficial or detrimental for H_2_ and CNT production is determined by the timing of water introduction. We show that water does not increase CMD rates under all conditions and propose that inconsistencies reported in the literature result from the distinct roles that water can play during the initial nanotube nucleation phase versus during stabilized CNT growth, which we aim to deconvolute here. Co-feeding water at varying partial pressures during the initial stage of catalyst evolution (*t* = 0 h) is found to decrease H_2_ production rates. In contrast, introducing water at various concentrations after catalyst stabilization (*t* = 3 h) can enhance methane conversion by over 400% at a water partial pressure of 24 Torr and 725 °C. Under stabilized conditions, water acts as a weak oxidizer, removing amorphous carbon from Ni and increasing the number of accessible active sites, thereby improving both hydrogen production and CNT yields. DFT results further indicate that the mechanism of carbon removal by water is dependent on the identity of adsorbed carbon species and that water is energetically capable of cleaning catalyst active sites, as proposed.

## 2 Methods

### 2.1 Catalyst Preparation

A 5 wt % Ni-25 wt % Mo over MgO catalyst was synthesized via the coprecipitation method. 1.1 g of Ammonium molybdate (99.99%, CAS:13106-76-8, ThermoFisher Scientific) and 0.5 g of nickel­(II) nitrate hexahydrate (98%, CAS:13478-00-7, Alfa Aesar) were sequentially dissolved in 20 mL of deionized water in a 100 mL beaker. The 100 mL beaker was placed inside a 250 mL beaker filled with approximately 150 mL of water to create a water bath to prevent local overheating. After the Ni and Mo precursors dissolved completely, 2.0 g of MgO powder (CAS: 1309-48-4, Spectrum Chemical) was introduced into the solution. The resulting mixture was continuously stirred and heated to a temperature range of 90–95 °C until complete evaporation of the water, yielding a damp solid powder. The solid was dried in a vacuum oven at 80 °C for 24 h. The obtained dry powder was calcined at 600 °C in an air flow of 100 sccm for 3 h. Images of Ni nanoparticles on the catalyst surface are available in Figure S1.

### 2.2 Reaction Measurements

Catalytic methane decomposition was performed with 100 mg of fresh catalyst loaded into a vertically oriented 1″ ID quartz tube reactor. A schematic of the reactor system is presented in Figure S2. 200 sccm of H_2_ was fed over the catalyst while increasing the reactor to 650 °C at 10 °C/min. At 650 °C, isothermal reduction in hydrogen was conducted for 30 min. After reduction, the reactor was purged with N_2_. The temperature was then adjusted to the desired growth temperature, ranging from 725 to 825 °C. Methane, along with a specified water concentration, was introduced into the reactor at the target growth temperature. Water was coinjected with methane using a syringe pump (KD Scientific). The inlet stream of the reactor was heated to a constant temperature of 104 °C to prevent water condensation. The nitrogen and methane flow rates were each set at 100 sccm after the reduction. Mass spectrometry (MKS Microvision Plus Residual Gas Analyzer) was used exclusively to monitor trends, including the transient response to initial water introduction at different water partial pressures, owing to its superior temporal resolution. All quantitative steady-state reaction rates were determined from GC measurements (GC-2014, Shimadzu Scientific Instruments), which provide higher accuracy for species quantification. Methane (CH_4_) was quantified using a flame ionization detector (FID), while hydrogen (H_2_), and carbon monoxide (CO) were quantified using a thermal conductivity detector (TCD). CMD rates were determined with respect to methane conversion, and example calculations are provided in the Supporting Information as Note S1. Carbon yield is reported on both a catalyst-mass basis (*g*_CNT_/*g*_cat_) and a nickel-mass basis (*g*_CNT_/*g*_Ni_). The latter was obtained by normalizing to the Ni loading in the catalyst (5%Ni–25%Mo/MgO). The average TOF values were also calculated in units of mol CH_4_ × mol Ni^–1^ × s^–1^ to provide a complementary, normalized metric supporting the yield values.

1 $$Yield\;of\;CNT\left(g_{CNT}/g_{cat}\right)=\frac{Mass\;of\;product\;\left(g\right)-Mass\;of\;catalyst\;\left(g_{cat}\right)}{Mass\;of\;catalyst\;\left(g_{cat}\right)}$$

2 $$Yield\;of\;CNT\left(\frac{g_{CNT}}{g_{Ni}}\right)=\frac{Yield\;of\;CNT\;\left(\frac{g_{CNT}}{g_{cat}}\right)}{0.05\left(\frac{g_{Ni}}{g_{cat}}\right)}$$

$$Average\;TOF=\frac{average\;mol\;{CH}_{4}\;converted}{mol\;Ni\times time\left(s\right)}=\frac{\frac{Yield\;of\;CNT\;\left(\frac{g_{CNT}}{g_{cat}}\right)}{12(\frac{g}{mol})}}{\frac{{0.05\times g}_{cat}\left(g\right)}{58.69\left(\frac{g}{mol}\right)}\times time\left(s\right)}$$ 3

### 2.3 CNT Characterization

#### 2.3.1 Transmission Electron Microscopy (TEM)

CNT diameter measurements were performed using a JEOL 2010 F TEM. The CNTs were sonicated for 10 min in ethanol and introduced dropwise onto lacey carbon films on 300 mesh copper grids purchased from Electron Microscopy Sciences (LC300-CU).

Scanning transmission electron microscopy (STEM) images were acquired using a JEOL Grand ARM microscope operated at 300 kV and equipped with a high-angle annular dark-field (HAADF) detector to determine the size of metallic Ni particles. Samples were deposited on lacey carbon–coated copper grids, and particle size distributions were obtained by measuring approximately 80 particles. Prior to imaging, the catalyst sample was reduced under H_2_ flow (100 mL/min) at 650 °C for 30 min at 1 atm.

#### 2.3.2 Scanning Electron Microscope (SEM-EDS)

The morphology of the samples was examined using a Thermo Fisher Scientific Quattro S Environmental Scanning Electron Microscope (ESEM). SEM observations were carried out at an accelerating voltage of 20 kV. Elemental analysis and mapping were performed using an energy-dispersive X-ray spectroscopy (EDS) detector integrated into the SEM system.

#### 2.3.3 Raman Spectroscopy

Raman spectra for all CNT samples, grown with and without water (no water injection, injection at *t* = 0 min, and *t* = 1 h), were collected at room temperature using a Renishaw InVia mapping Raman microscope with a 532 nm green laser set to 5%. Data acquisition and processing were performed using Wire 4.1 software. The data was normalized, and peak areas were obtained using Origin after performing normalization and baseline correction.

#### 2.3.4 Thermogravimetric Analysis (TGA)

Thermogravimetric analysis was performed using a NETZSCH STA Jupiter 449 F1 thermogravimetric analyzer with a constant argon flow of 40 mL/min. For each experiment, 50 mg of CNTs were loaded into an alumina crucible. The samples were heated in 250 mL/min of air to 850 °C at a rate of 5 °C/min and then maintained at this temperature for 3 h.

### 2.4 DFT Calculations

All density functional theory (DFT) calculations were performed with the Vienna Ab initio simulation package (VASP)^34^ using the Perdew–Burke–Ernzerhof (PBE) exchange–correlation functional within the generalized gradient approximation (GGA).^35^ A semiempirical DFT-D3 dispersion scheme was applied to account for the van der Waals interactions present in the system. Ionic cores were described by the projector-augmented wave (PAW) method with *a* plane wave cutoff energy of 400 eV.^36^ All transition states (TS) were determined using the nudged elastic band (NEB) method,^37^ with vibrational frequency analysis conducted to validate the TS. Thermodynamic variables (i.e., internal energy, zero-point energy, enthalpy, and entropy) were computed using statistical thermodynamics.^38^ Specifically, we applied the quasi-rigid rotor harmonic oscillator (q-RRHO) method to calculate entropies from low-lying modes,^39^ as described in the literature and demonstrated to better capture the entropic contribution of small, loose surface-bound species.^40^

Although both Ni and Mo are present in the catalyst, existing literature indicates that Mo/MgO exhibits negligible activity for methane decomposition under comparable conditions, whereas Ni is the active metal responsible for the reaction.^41^ Our previous results have similarly shown that under these conditions, Mo/MgO contributes negligible activity during this reaction.^12^ In the bimetallic Ni–Mo/MgO catalyst, the primary role of Mo is to stabilize the Ni particles, without directly participating in methane activation.^42,43^ Therefore, the Ni(111) surface, the most stable and abundant crystallographic structure of nickel,^44,45^ was employed as the model system in this study. While we acknowledge that the real catalyst under operating conditions will be more complex than the ideal surfaces utilized in the DFT simulations reported here, the general trends with respect to the role of water interaction with surface species should still hold. Given the experimental conditions, where surface carbon tends to diffuse into the bulk, we extended our investigation to include the nickel carbide phase. This model system was constructed with four carbon atoms positioned beneath the first Ni(111) surface layer (corresponding to Ni/C = 8:1), as shown in Figure S3. A periodic slab with a vacuum region of 15.0 Å was used to separate the periodically repeated slabs. Four-layer models were used, where the top two layers were relaxed, while the bottom two layers were fixed at their bulk positions. A 3 × 3 × 1 *k*-point mesh was utilized for the slab calculations. Because the exact carbon concentration under reaction conditions is unknown, the present work does not aim to determine the precise surface composition. Instead, by comparing pristine Ni(111) and the Ni_8_C model, we focus on elucidating the effect of H_2_O and its role in promoting carbon removal on both Ni and NiC surfaces.

### 2.5 *n*-Hexane Reactions

Gas-phase reactions of *n*-hexane were conducted in a fixed-bed 1-in. quartz reactor under atmospheric pressure. The reactor was equipped with a K-type thermocouple positioned at the center of the catalyst bed to ensure accurate temperature control. A total of 50 mg of 5%Ni–25%Mo/MgO catalyst was packed between quartz wool plugs and carburized for 5 min under the same conditions used for CNT growth. Following carburization, the reactor was cooled to 300 °C for *n*-hexane reaction studies conducted under three different conditions. In the first condition, *n*-hexane (Sigma-Aldrich, ReagentPlus, ≥99%) was fed at a liquid flow rate of 0.5 mL h^–1^ over the carburized catalyst. In the second condition, the catalyst was exposed to distilled water (0.1 mL h^–1^) for 15 min, followed by a 15 min helium purge prior to introducing *n*-hexane at 0.5 mL h^–1^. In the third condition, *n*-hexane (0.5 mL h^–1^) and water (0.1 mL h^–1^) were cofed over the carburized catalyst. Both water and *n*-hexane were introduced using KDS Series 100 syringe pumps. Reaction products were analyzed using a Shimadzu QP-2010 GC–MS/FID system equipped with an HP-PLOT Al_2_O_3_ S capillary column connected directly to the reactor outlet.

## 3 Results

### 3.1 Water Impacts during the Initial Moments of CMD

Figure 1 illustrates the hydrogen evolution over time during CNT growth, where methane is cofed with varying water concentrations over a 5%Ni–25%Mo/MgO catalyst. This allows for assessment of water incorporation effects at different partial pressures during the initial CNT growth stage. As shown in Figure 1a, water introduction at the beginning of CMD (*t* = 0 h) negatively impacts both the maximum and steady-state hydrogen production rates for water partial pressures between 0 to 24 Torr. We hypothesize that water incorporation impedes the carburization process, an essential step for forming the Ni particles necessary for CNT nucleation. Our previous study showed through XPS and XRD that a NiMoO_4_ phase, formed during reduction, is rapidly transformed into Mo_2_C upon methane introduction, causing exsolution of active Ni particles. This has been explained in more detail in our prior study.^12^ Therefore, the presence of water could hinder the formation of Mo_2_C by stabilizing Mo-based oxides or hydroxides, disrupting nucleation of Ni particles.^46,47^ The reduced rate of Mo_2_C formation would decrease rates of metal particle exsolution from the underlying mixed oxide, leading to entrapped Ni atoms that fail to exsolve. Some authors have also proposed that Mo_2_C serves as a carbon reservoir that prevents rapid encapsulation of Ni particles, facilitating CNT growth.^48,49^

**Figure 1:**
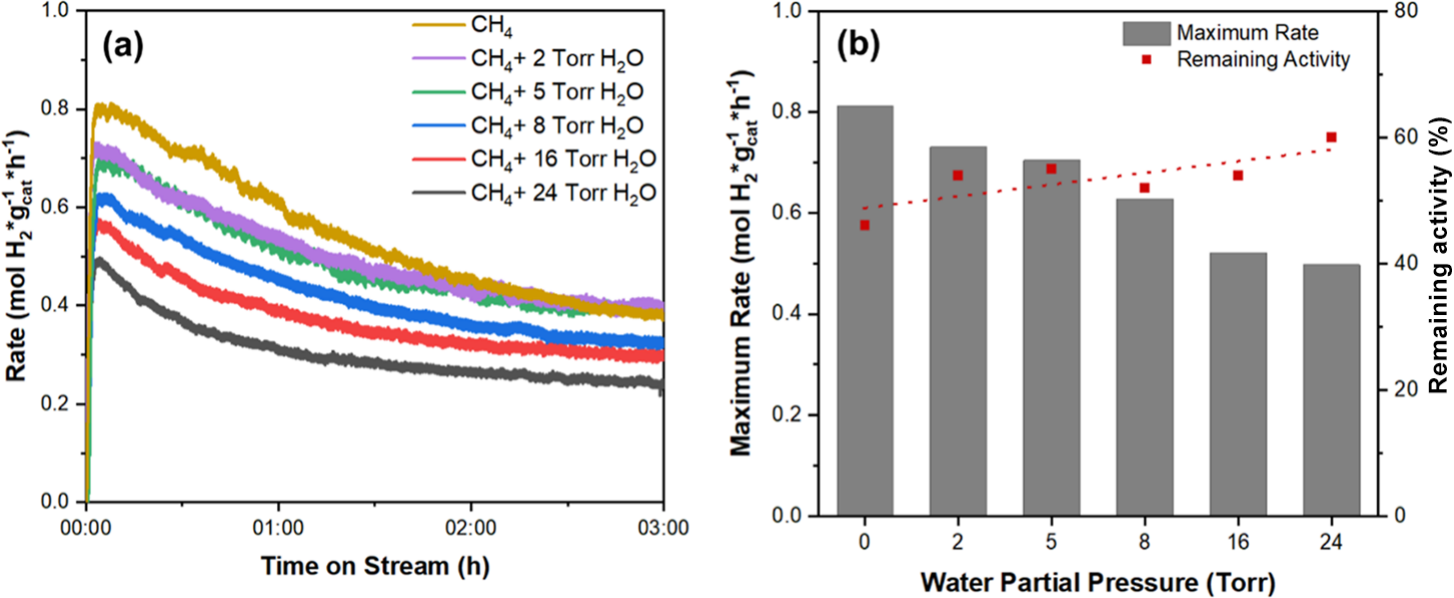
Impact of water concentration on CNT growth and catalyst stability (a) H_2_ production rate when cofeeding various water concentrations with methane at *t* = 0 h over Ni–Mo/MgO (b) The maximum rate of hydrogen production and remaining activity of the catalyst at *t* = 3 h. $\left(Remaining\;activity=\frac{\left[Rate\;at\;3hr\right]}{\left[\mathrm{Max}\;rate\right]}\right)$ . Reaction conditions for all reactions were: *P*_Total_ = 1 atm, Flow of CH_4_ = 100 mL/min, *W*_cat._ = 100 mg, and *T*_rxn_ = 800 °C. The catalyst was previously reduced in H_2_ flow (200 mL/min) at 650 °C for 30 min.

Although the maximum H_2_ production rates in Figure 1a decline as water concentration increases, Figure 1b indicates that a greater proportion of the maximum rate for each individual trial is maintained after 3 h with higher water partial pressures. Carbon yields and TOF values for each trial are presented in Table S1. Estimated average TOF values from selected literature studies are also summarized in Table S4 to provide additional context for benchmarking catalytic activity. Our results suggest that water suppresses CMD rate by hindering the exsolution of Ni particles on the Ni–Mo/MgO catalyst. However, the increased proportion of sustained activity indicates that water may play a role in catalyst stability. Futaba et al. captured differing effects of water on single-walled CNT synthesis from ethylene on Fe/Al_2_O_3_, claiming that optimal water concentrations increased both CNT growth rates and catalyst lifetimes.^33^ In contrast, the decreased CMD rates and extended catalyst lifetimes reported here may be attributed to differences in carbon chemical potential and relative reaction rates of methane activation versus particle encapsulation in the presence of water. Furthermore, ethylene is more easily activated than methane, likely facilitating catalyst carburization even in the presence of cofed water. This difference in CNT growth behavior in the presence of water can likely be attributed to differences in the hydrocarbon feedstock. Thus, we hypothesize that incorporating water during CMD over Ni–Mo/MgO at the initial stage decreases the carbon chemical potential on the catalyst surface, causing decreased Ni exsolution while better maintaining the activity of exsolved Ni particles long-term.

To further understand the impact of water incorporation on CNT structure, the produced CNTs were characterized by thermogravimetric analysis (TGA) and transmission electron microscopy (TEM). Figure 2a presents TGA results, showing that the synthesized CNTs begin to oxidize around 550 °C, indicating a predominantly defect-free structure. The total weight loss was 94% for the water-free CNTs and 91% for water-*co* fed CNTs; this further proves that carbon yields decreased due to water incorporation. The noncombustible mass results from the catalyst, which remains attached to the CNTs. While TGA mass loss slopes can be subject to diffusion limitations resulting from CNT packing,^50^ we attribute the different slopes observed here to differences in carbon defect density, which will be addressed later. TEM images presented in Figure 2b,c show comparable CNT structures, with average outer diameters of 15 ± 5 nm for the water-free CNTs and 14 ± 5 nm for the water-*co* fed CNTs, where the associated error is one standard deviation. This result indicates that the addition of water mainly affects the reaction efficiency rather than the catalyst particle size distribution. This interpretation is supported by previous studies showing that the diameter of carbon nanotubes correlates with the diameter of the catalyst particle that produced the nanotube.^51^ Histograms depicting CNT diameter distributions are provided in Figure S4 and were obtained based on Figures S5 and S6.

**Figure 2:**
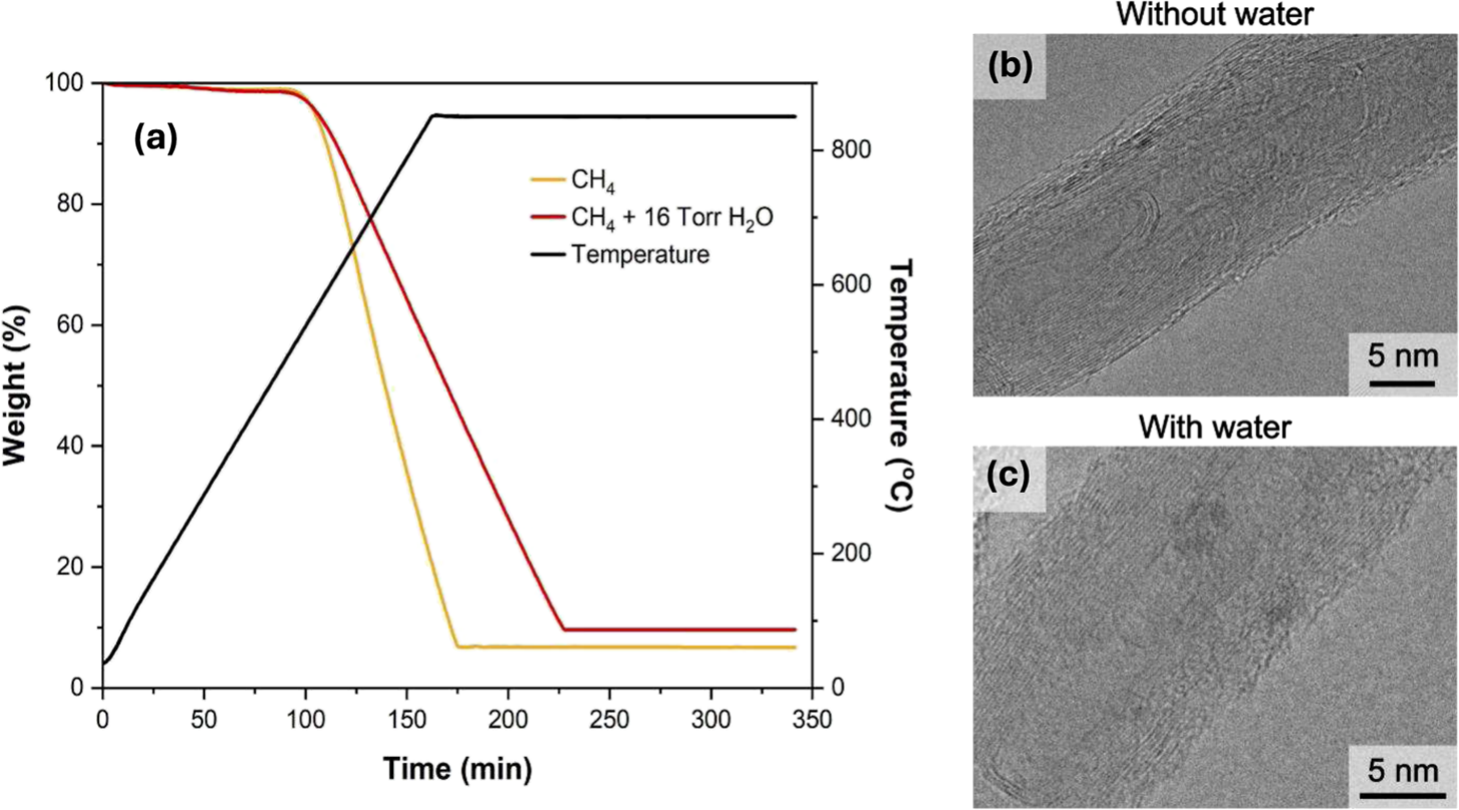
Characterization of CNTs synthesized with water during the initial stage of the growth. (a) TGA comparing CNTs grown with and without water (b) TEM analysis of CNTs produced without water and (c) with 16 Torr H_2_O. The materials were prepared under the following conditions: *P*_Total_ = 1 atm, Flow of CH_4_ = 100 mL/min, *W*_cat_ = 100 mg, *t*_rxn_ = 3 h, and *T*_rxn_ = 800 °C. The catalyst was prereduced in H_2_ flow (200 mL/min) up to 650 °C for 30 min.

As discussed, since water does not appear to modify CNT or nanoparticle diameter, the observed decrease in carbon yield supports the hypothesis that fewer active Ni sites exsolve from the catalyst as water partial pressure increases during the initial stage of CNT growth. Furthermore, consistent with observations typically associated with base-growth in the absence of water, CNTs formed in the presence of water exhibit similar features, with very few nanoparticles observed inside the CNTs, trapped within the tube interior, or located at the CNT tips. Specifically, metal particles were observed in approximately 5% of CNTs grown without water cofeeding, compared to over ∼10% when water was introduced. While this indicates that water may increase metal loss among CNTs, the base-growth mechanism remains dominant under both conditions.

### 3.2 Water Impacts after CMD Stabilization

Because water can alter the carburization process and impact catalyst morphology, as previously discussed, assessing the role of water on methane activation kinetics requires a consistent prestabilization step. This ensures that a comparable number of Ni nanoparticles of comparable diameter exsolve across all trials, facilitating assessment of CMD kinetics during water incorporation independent of catalyst morphology. Thus, 3 h of CNT growth at 800 °C was established as the stabilization procedure, with water introduced at various concentrations after those 3 h. At this stage, Ni exsolution from the molybdenum carbide support is complete, ensuring that subsequent comparisons are made on catalysts with fully developed and comparable active phases. This approach also enables a direct comparison between the effect of water on a carbon-free catalyst surface (*t* = 0 h) and a carbon-rich surface at steady state (*t* = 3 h). This condition isolates the role of water on carbon-covered Ni surfaces, minimizing impacts from structural evolution or further rapid deactivation that could otherwise complicate the interpretation of kinetic trends.

Although the reaction was allowed to proceed for 3 h to approach steady-state behavior, complete stability cannot be assumed, as some degree of deactivation is unavoidable for any metal catalyst. The measured CMD rate is a function of methane activation and catalyst deactivation, as shown in eq 4.

4 $$r_{measured}=f(r_{{CH}_{4}},\varphi \left(t\right))$$

where *r*_CH_4__ represents the rate of methane consumption and φ­(*t*) represents activity loss as a function of time.

Thus, a mathematical normalization step is necessary to produce the corrected CMD rate in eq 5. This ensures that reported rates reflect intrinsic catalytic activity and are not convoluted by time-dependent decay. This normalization calculation is produced by fitting polynomial coefficients to CMD rates measured in the absence of water, as shown in Table S2, to account for deactivation. The detailed calculations and a representative example are provided in Note S1.

5 $$r_{corrected}=f\left(r_{{CH}_{4}}\right)$$

Figure 3 illustrates the corrected H_2_ production rates when introducing water after CNT growth stabilization at 800 °C. The corrected CMD rates indicate that water incorporation facilitated definitive CMD rate increases of 7%, 30%, and 47%, at water partial pressures of 8, 16, and 24 Torr, respectively, compared to the baseline H_2_ production rate in the absence of water. Notably, water cofeeding induces a temporary enhancement in catalytic activity; however, upon removal of water, the reaction rate returns to its original baseline, indistinguishable from that observed under non-water conditions. This reversible behavior indicates that water cofeeding does not lead to long-term structural modifications of the catalyst, but instead affects the reaction through short-lived, surface-mediated processes. The rate profile as a function of time is available in Figure S7.

**Figure 3:**
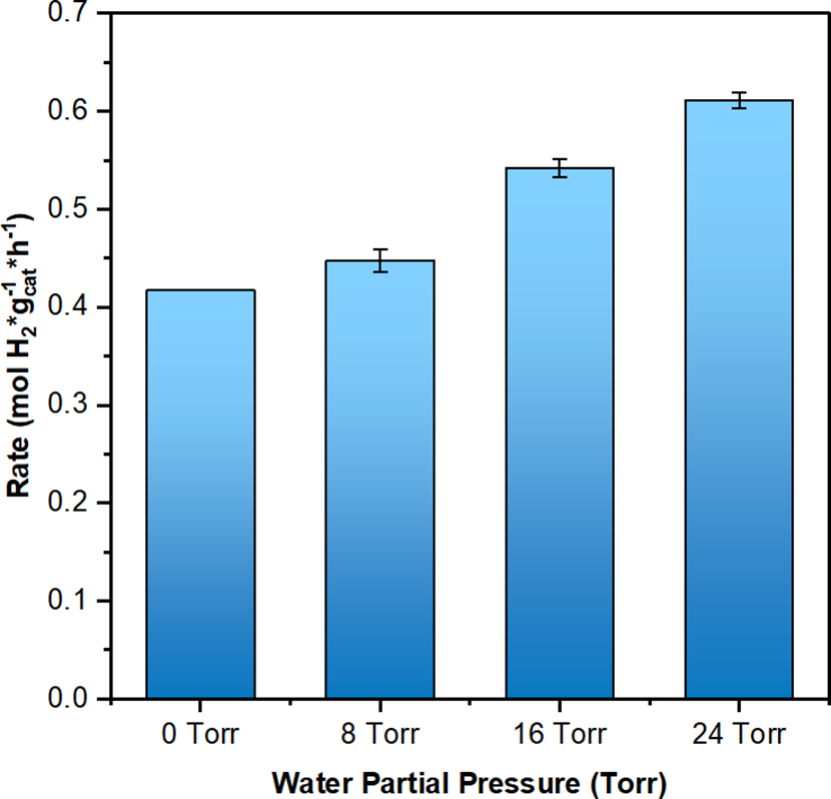
Effect of water introduction on corrected H_2_ production rates after catalyst stabilization. Reaction conditions for all reactions were: *P*_Total_ = 1 atm, Flow of CH_4_ = 100 mL/min, *W*_cat_. = 100 mg, and *T*_rxn_ = 800 °C. The catalyst was previously reduced under H_2_ flow (200 mL/min) at 650 °C for 30 min.

To elucidate the origin of this rate increase, it is necessary to consider the alternative reactions presented in Table 1 that may occur upon introducing water into the system. Water introduction during CMD promotes multiple parallel reactions, with hydrogen resulting from methane via methane decomposition (Rxn. 1) as well as from water through the water–gas (Rxn. 2) and water–gas shift (WGS) reactions (Rxn. 3).^52,53^ These alternative reactions could potentially remove carbon from Ni active sites via Rxn. 2, making active sites accessible for additional CMD turnovers that facilitate increased methane conversion. It should be clarified that reported CMD rates are calculated from methane conversion and therefore are not convoluted by hydrogen produced in alternative reactions. These results align with those of Yoshihara et al., who reported that water maintains catalyst activity during CNT growth over Fe–Mo/MgO by removing amorphous carbon and regenerating active sites.^54^

**Table 1:** Possible Reactions During CMD in the Presence of Water^52,53^

CH_4(g)_ → C_(s)_ + 2H_2(g)_	Methane Decomposition (Rxn.1)
H_2_O_(g)_ + C_(s)_ → CO_(g)_ + H_2(g)_	Water–Gas Reaction (Rxn.2)
H_2_O_(g)_ + CO_(g)_ ↔ CO_2(g)_ + H_2(g)_	Water Gas Shift Reaction (Rxn.3); K_e_ ^56^
2CO_(g)_ → C_(s)_ + CO_2_	Boudouard Reaction (Rxn.4)

Although the water–gas shift (Rxn. 3) and Boudouard reactions (Rxn. 4) may, in principle, occur under these conditions, we treat their contributions to both hydrogen generation and carbon consumption reactions as in thermodynamic equilibrium, highlighting that the kinetic relevance of these reactions relative to direct CMD is expected to be negligible. Only a small amount of water (1–3 mol %) was cofed with methane, and analysis indicates that at all temperatures, the vast majority of the introduced water (>95%) is converted to CO. The remaining oxygen is distributed between H_2_O and CO_2_ according to the WGS equilibrium, resulting in CO_2_ partial pressures on the order of 10^–4^ atm. At such low CO_2_ concentrations and elevated temperatures (>700 °C), the Boudouard reaction is thermodynamically unfavorable and is therefore not expected to contribute meaningfully relative to catalytic methane decomposition.^55^ Therefore, under these conditions, the observed rates primarily reflect CMD kinetics.

### 3.3 Temperature Effects on Water Incorporation after CMD Stabilization

At the tested water partial pressures, water incorporation at 800 °C was found to increase CMD rates, even after correcting for catalyst deactivation. Upon introduction, water may catalytically decompose into OH and H species over Ni metal, Mo_2_C, or Mo metal sites, potentially modifying the surface coverage and promoting competitive reactions with carbon species.^57–60^ The coverage of such species is likely temperature-dependent and could dictate the role of water during CMD. For this reason, the role of water was investigated across different temperatures.

Figure 4a illustrates the baseline and corrected CMD rates obtained from 725 to 800 °C with 16 Torr H_2_O introduced after stabilization, where the baseline rate is the CMD rate in the absence of water incorporation. Graphs of the corrected rates for these trials are found in Figure S7.

**Figure 4:**
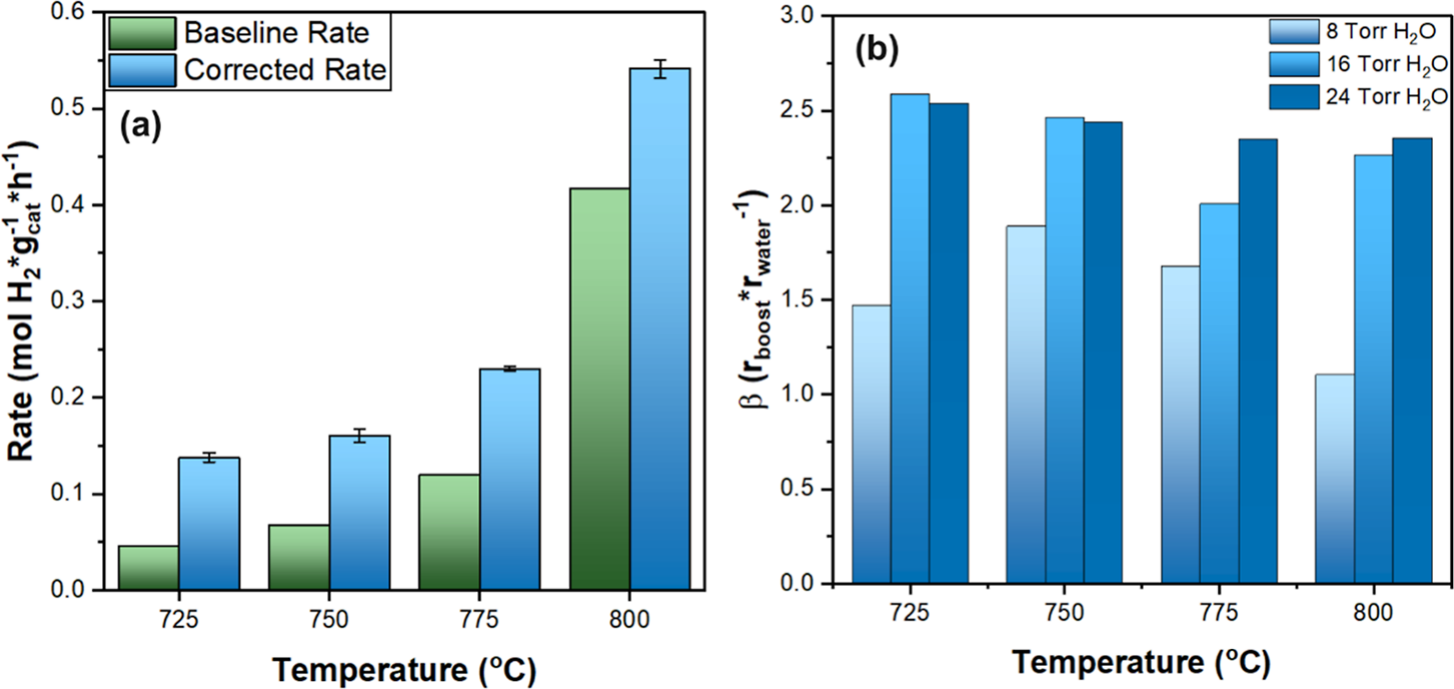
Temperature effects on water introduction after CMD stabilization. (a) H_2_ generation rates measured at 16 Torr H_2_O as a function of temperature, and (b) The ratio β = *r*_boost_/*r*_water_ as a function of temperature and water partial pressure. Reaction conditions to achieve stabilization for all reactions were: *P*_Total_ = 1 atm, Flow of CH_4_ = 100 mL/min, *W*_cat_. = 100 mg, *t*_rxn_ = 3 h, and *T*_rxn_ = 800 °C. The catalyst was previously reduced under H_2_ flow (200 mL/min) at 650 °C for 30 min. Temperature (725–800 °C) and water partial pressure (0–24 Torr) were modified after achieving stabilization (*t* = 3 h).

Temperature could influence (i) the rates of methane activation and, consequently, the relative rates of catalyst encapsulation, and (ii) the involvement of surface-bound oxygen species, which may exist at different coverages and affect C–H bond activation.^61,62^ However, across the range of temperatures investigated, the introduction of water caused comparable enhancements in methane conversion, indicating that its promotional effect is largely independent of temperature. This observation suggests that the effect of water is not strongly temperature dependent and likely arises from surface-mediated processes rather than altering the intrinsic thermal kinetics of methane activation.

To better understand the impact of water on CMD, Figure 4b presents β, a metric that represents the ratio between the boost in the CMD rate due to water incorporation and the rate of water consumption, providing mechanistic insight into the role of water during CMD. This metric is presented in eq 6.

6 $$\beta =\left(\frac{r_{corrected}-r_{baseline}}{r_{water}}\right)=\frac{r_{boost}}{r_{water}}$$

By this expression, a β value of 2 mol H_2_ per mol H_2_O represents the stoichiometric upper limit for water enabling a single methane turnover directly through the water gas reaction (Rxn.2), cleaning the catalyst surface to regenerate Ni active sites. For example, if one water molecule removes one C atom bound to the Ni surface, as described in Rxn. 2, it can regenerate one Ni active site. The newly accessible active site can facilitate a single CH_4_ turnover, releasing 2 H_2_ molecules before deactivating again by strongly binding the single C atom from the CH_4_. Values of β greater than 2 mol H_2_/mol H_2_O result from nonstoichiometric interactions with water, indicating that water does not solely facilitate the water gas reaction. Rather, β > 2 indicates that active sites regenerated by water promote more than a single turnover before strongly binding a C atom and becoming inactive again.

Thus, Figure 4b depicts that β > 2 at 16 and 24 Torr of H_2_O but β < 2 at 8 Torr. The lower β value at 8 Torr can be rationalized by the fact that this was the first sequential introduction of water after approaching steady state CNT synthesis. Therefore, the catalyst surface was likely covered with substantial amounts of carbon; the small initial dose of H_2_O was likely insufficient to fully regenerate active sites or establish a potential OH/O population, limiting the number of CH_4_ turnovers enabled per H_2_O molecule. After this initial conditioning step, higher water doses (16 and 24 Torr) produced β > 2, consistent with water acting to regenerate sites or promoting C–H activation once the Ni surface is accessible. These values of β indicate that water facilitates more than a single additional methane turnover per water molecule consumed, independent of temperature and, beyond a minimum required value of water partial pressure.

Importantly, β values exceeding two provide mechanistic constraints on the possible role of oxygen-containing Mo species, which have been proposed to exhibit catalytic activity toward methane activation.^63^ Several studies have indicated that the formation of bulk MoC_*x*_O_*y*_ phases is thermodynamically unfavorable under the methane- and carbon-rich environments.^64,65^ Lamont et al. demonstrated that Mo_2_C remains largely stable under hydrocarbon-rich environments, with significant oxidation occurring only under highly oxidizing conditions.^64^ These reports indicate that if MoC_*x*_O_*y*_ species are formed, it is likely through transient surface decoration of the Mo_2_C surface with oxygen- or hydroxyl-containing species, while retaining an underlying carbide framework. Consistent with this interpretation, XRD results (Figure S8) show no significant structural differences between samples exposed to methane or methane with 16 Torr of water. Sullivan et al. similarly report no change in bulk structure via XRD and the formation of transient surface species.^66^

The formation of transient surface MoC_*x*_O_*y*_ species that contribute to methane activation would facilitate stoichiometric boosts in methane conversion, and therefore a β value of 2. In this case, every oxygen from water would create an oxycarbide structure, and each of those species would activate a CH_4_ molecule, yielding CO or H_2_O, H_2_, and a regenerated molybdenum carbide structure. The values reported here, where β > 2, indicate increases in methane conversion beyond stoichiometric values. Thus, we hypothesize that water directly facilitates the regeneration of Ni active sites that otherwise bind C and are inactive for methane conversion, as this can explain β values greater than two, where activation on an oxycarbide cannot. While the formation of surface MoC_*x*_O_*y*_ species is possible, we conclude that if such species do form, they are not solely responsible for the boosts in methane conversion reported here and do not alter the conclusions of this work.

Raman spectroscopy was conducted to quantify the D/G intensity ratio shown in Figure 5. The intensity ratio of the D-band (∼1350 cm^–1^) to the G-band (∼1580 cm^–1^) is commonly used to represent the defect density of CNTs.^67^ As shown in Figure 5, CNTs grown in the presence of water, regardless of when it is introduced, exhibit a lower D/G ratio, indicating fewer structural defects compared to samples without water incorporation. As a result, water influences the morphology of the CNT through the removal of carbon deposits on active sites prior to graphitization. It can be concluded that water incorporation can reduce the defect density of CNTs.

**Figure 5:**
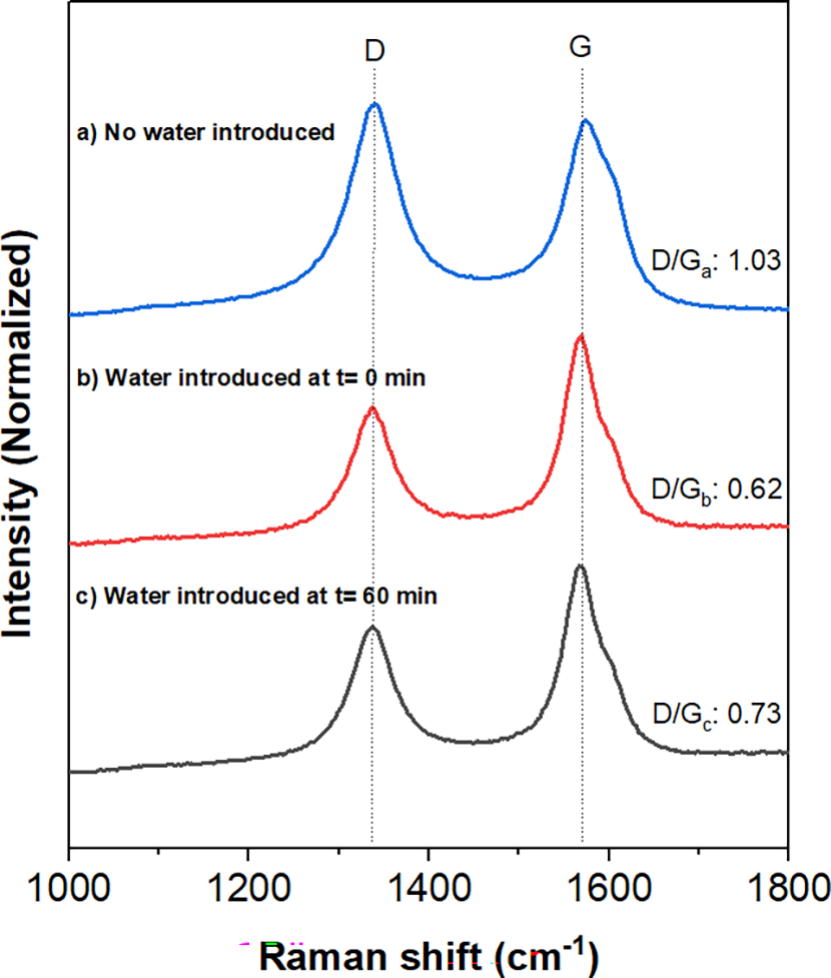
Raman spectroscopy of CNTs grown with and without water. (a) CNTs without water, (b) CNTs with water introduced at *t* = 0 min, (c) CNTs with water introduced at *t* = 60 min. Conditions: *P*_Total_ = 1 atm, *T*_rxn_ = 800 °C, *W*_cat_ = 100 mg, *F*_CH_4__ = 100 mL/min. All samples were previously reduced with H_2_ at 650 °C for 30 min.

Figure 6 shows that upon water incorporation, the exponential influence of temperature on reaction rate, typically referenced as the apparent activation energy, for water-free and water-assisted CMD is reduced from 137 ± 11 kJ/mol to 45 ± 14 kJ/mol, respectively. Figure S9 depicts methane conversion data used to calculate reported activation energies. We hypothesize that water introduction after catalyst stabilization decreases the apparent activation energy by inhibiting the deactivation of metal surface sites that possess lower inherent methane activation energies. For instance, the activity of a step-edge Ni site, which is known to be highly active for methane decomposition due to its undercoordinated nature, facilitates strong binding of carbon species and, therefore, more rapid deactivation.^68,69^ However, water introduction could potentially suppress deactivation by cleaning these sites and modifying the relative ratio of CMD turnovers on highly active step-edges versus on standard Ni planes, thereby increasing the relative contribution of the cleaned sites to the overall CMD rate. Abild-Pedersen et al. have shown that CMD on a step-edge site proceeds with a lower activation energy than on less active plane sites.^68,69^ Thus, the measured decrease in apparent activation energy likely reflects a greater contribution from these more active sites. This is further supported through DFT analysis discussed later. Importantly, this reduction in apparent activation energy does not suggest a shift in the rate-determining step but rather reflects the removal of convoluting factors, providing a more accurate representation of the activation energy for methane decomposition.

**Figure 6:**
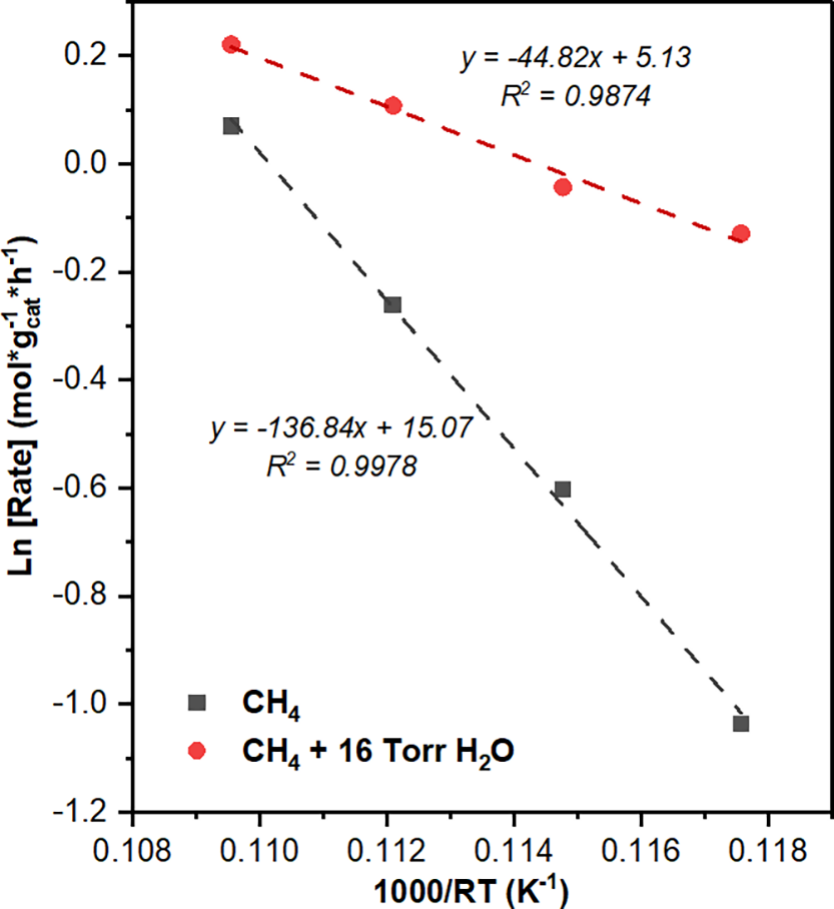
Apparent activation energies for poststabilization CMD with and without water incorporation. Reaction conditions to achieve steady state for all reactions were: *P*_Total_ = 1 atm, Flow of CH_4_ = 100 mL/min, *W*_cat_. = 25 mg, and *T*_reaction_ = 750–825 °C. The catalyst was previously reduced under H_2_ flow (200 mL/min) up to 650 °C for 30 min. Temperature was varied after achieving steady state (*t* = 3 h).

Although the activation energies reflect the apparent values, it is also necessary to assess whether transport phenomena could contribute to the measured kinetics. Accordingly, CH_4_ conversion was analyzed as a function of time-on-stream at all investigated temperatures and the same H_2_O partial pressure, as shown in Figure S9. Our results indicate that methane activation rate is significantly higher during the initial stages of carbon growth, followed by a transition to a comparatively stable regime after 3 h on stream, which is used for kinetic analysis. Methane diffusion to the catalyst surface is sufficient to achieve initial reaction rates that are significantly faster than those reported for the steady state kinetic regime. This observation indicates that external and internal mass-transfer limitations are unlikely to constrain the measured rates under the conditions used for kinetic evaluation. Unlike conventional reactions, where particle-size variation can be used directly to probe diffusion effects, in CMD such changes also inevitably modify the average coordination environment of exposed metal sites and the metal–carbon interface, thereby directly affecting the intrinsic rate of methane activation. To further substantiate the kinetic relevance of the data, internal diffusion criteria were additionally evaluated and are provided in the Supporting Information (Note S2).

### 3.4 Proposed Role of Water during CMD

Throughout CMD, methane decomposes into C and H atoms on the surface of the Ni–Mo/MgO catalyst, as shown in Rxn. 1. The freshly generated C atoms rapidly dissolve into molybdenum, leading to the formation of Mo_2_C. Previous results revealed that Mo_2_C formation disrupts Mo–Ni interactions, promoting Ni particle exsolution to facilitate CNT growth^12^ as shown in Figure 7a. However, Mo species have a high affinity for oxygen;^70^ therefore, the presence of water at the beginning of the reaction could potentially induce competition between molybdenum oxide (MoO_3‑x_) and molybdenum carbide (Mo_2_C) formation. We highlight the oxidative impact of water on a Mo_2_C surface by revealing how water introduction, even at milder conditions can decrease C–H bond activation of alkanes in Figure S10. This competition could reduce the extent of Ni particle exsolution, creating fewer active sites for CNT growth and facilitating decreased H_2_ production. Figure 7b illustrates the exsolution of fewer Ni active sites on the catalyst surface due to Mo-oxidation resulting from water incorporation before molybdenum carbide formation.

**Figure 7:**
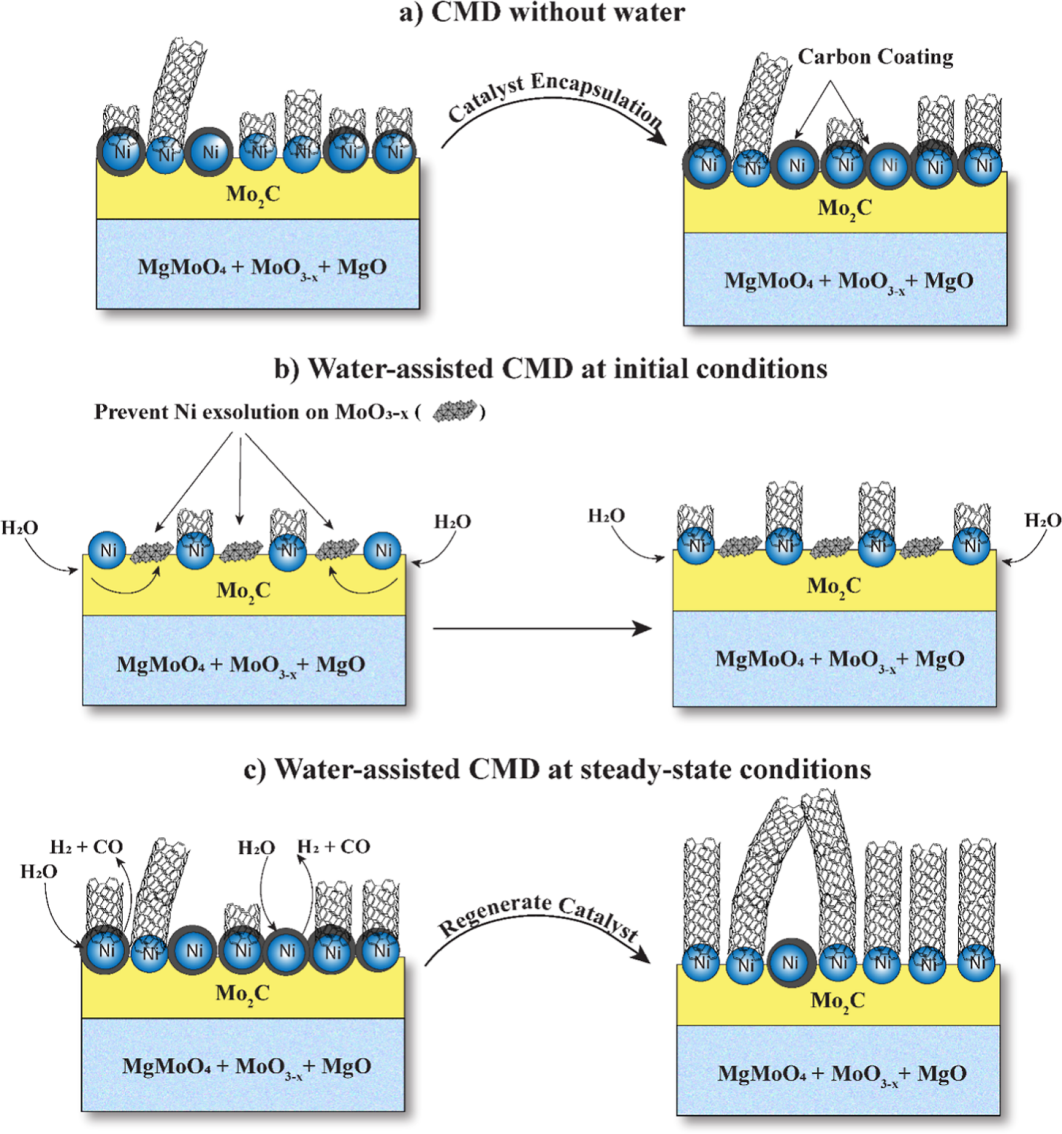
Schematic of the impact of cofed water on catalyst surface modification during CMD. (a) carbon nanotube growth pathway in the absence of water; (b) pathway with water at the initial stage; (c) pathway with water at the steady stage.

In the absence of water, accumulation of amorphous carbon on the catalyst surface increases with time, progressively encapsulating active sites and reducing catalytic activity, as depicted in Figure 7a, until approaching a steady state rate (*t* = 3 h). At this stage, water introduction does not decrease CMD rates because it no longer facilitates the proposed competition between molybdenum oxide and molybdenum carbide formation that limits Ni particle exsolution. Rather, after catalyst stabilization, water removes amorphous carbon from the catalyst surface, reducing the CNT defect density, regenerating Ni active sites, and enhancing CMD rates (Figure 7c). The energetics of the cleaning effect of water are further investigated with DFT.

### 3.5 Mechanistic Insights into Water-Assisted CMD

The interaction of water with carbon species at the surface is complex due to multiple parallel reactions occurring simultaneously, as described in Table 1. DFT calculations were performed to gain deeper insight into how water interacts with the saturated carbon catalyst support. The dissociation pathways of H_2_O into surface OH, H, and O species were examined on two different surfaces, Ni(111) and Ni(111) with four carbon atoms incorporated into the first lattice layer (Ni_8_C) as shown in Figure S3. The activation energies of the two water dissociation pathways are shown in Figure S11. The results reveal that the carbide layer increases activation barriers for both H_2_O and OH dissociation. While H_2_O readily dissociates on both surfaces, the higher barriers on Ni_8_C suggest a destabilizing effect from Ni carbide.

When amorphous carbon deposits on the catalyst surface, H_2_O is expected to play an additional role by promoting the removal of this deposited surface carbon, as has been supported by others. Given the consistent formation of carbide, Ni_8_C was selected as the model for subsequent calculations. Under reaction conditions, where the surface is expected to be substantially covered with carbon, various mechanisms for water dissociation were investigated, with particular emphasis on interactions between water and surface-bound carbon species. One proposed mechanism involves carbon-assisted water dissociation, whereas an alternative pathway considers initial H_2_O dissociation to form atomic O, which subsequently reacts with surface carbon.

Two distinct carbon structures on the nickel carbide catalyst surface were evaluated: a single carbon atom and a carbon trimer, selected to represent possible carbon species present on the surface species under reaction conditions. A C adatom and a C_3_ trimer were selected as representative structures because they reflect the fundamental processes in carbon nucleation on metal surfaces: individual carbon adsorption and the initial formation of small carbon clusters. The detailed pathways for these mechanisms are presented in Note S4.

Figure 8 shows the energy profile of H_2_O dissociation on nickel carbide with a single carbon atom and a carbon trimer structure at 1073K, corresponding to the experimental conditions. As shown in Figure 8a, the carbon-assisted water dissociation mechanism suggests that H_2_O preferentially reacts directly with C on the surface to form the COH* intermediate and H atom, which then undergoes dehydrogenation to produce CO. The activation barriers for these steps are 103 and 47 kJ/mol, respectively. This reaction pathway is both thermodynamically and kinetically more favorable compared to the alternative pathway, where H_2_O first dissociates, followed by the interaction between C* and O* as shown in Figure 8b. The later pathway has a significantly higher activation barrier of 151 kJ/mol, due to the strong adsorption of oxygen on the Ni active site, preventing other oxygenated species, such as CO, from interacting with the Ni active surface due to competitive adsorption. However, the reaction mechanism for C_3_ and H_2_O differs significantly. For the carbon-assisted H_2_O dissociation pathway, the direct attack of water onto the C_3_ trimer to form the C_3_OH* intermediate requires a high activation energy of 136 kJ/mol, and to further form C_2_* and COH*, an activation energy of 81 kJ/mol is needed. In contrast, the carbon oxidation by surface oxygen from H_2_O dissociation mechanism is more favorable. Here, water first dissociates on the surface to release an oxygen atom. This oxygen species then interacts with the C_3_ trimer to form the OC_3_ intermediate, which requires a lower activation energy of 82 kJ/mol. Subsequent breaking of the C–C bond to form a C=C double bond and CO requires an activation energy of 70 kJ/mol. This indirect pathway is also thermodynamically more favorable, with reaction energies of −104 kJ/mol for the formation of OC_3_* and −51 kJ/mol for the formation of C_2_* + CO*. This suggests that the indirect pathway, involving initial dissociation of H_2_O and subsequent reaction with the released oxygen, is both kinetically and thermodynamically more efficient. Note that water adsorption becomes less favorable at high temperatures. The Gibbs free energy change for water adsorption was estimated by q-RRHO method. The Δ*G* for water adsorption is approximately 30–130 kJ/mol at the reaction temperature. This large uncertainty arises from the estimation of the entropy of adsorbed water, which is assumed to retain either 2/3 or 1/3 of the gas-phase water entropy.^71^ The gas-phase water molecule has an entropy of 256 J/mol·K at the reaction temperature, calculated using the Shomate equation with parameters from the NIST database.

**Figure 8:**
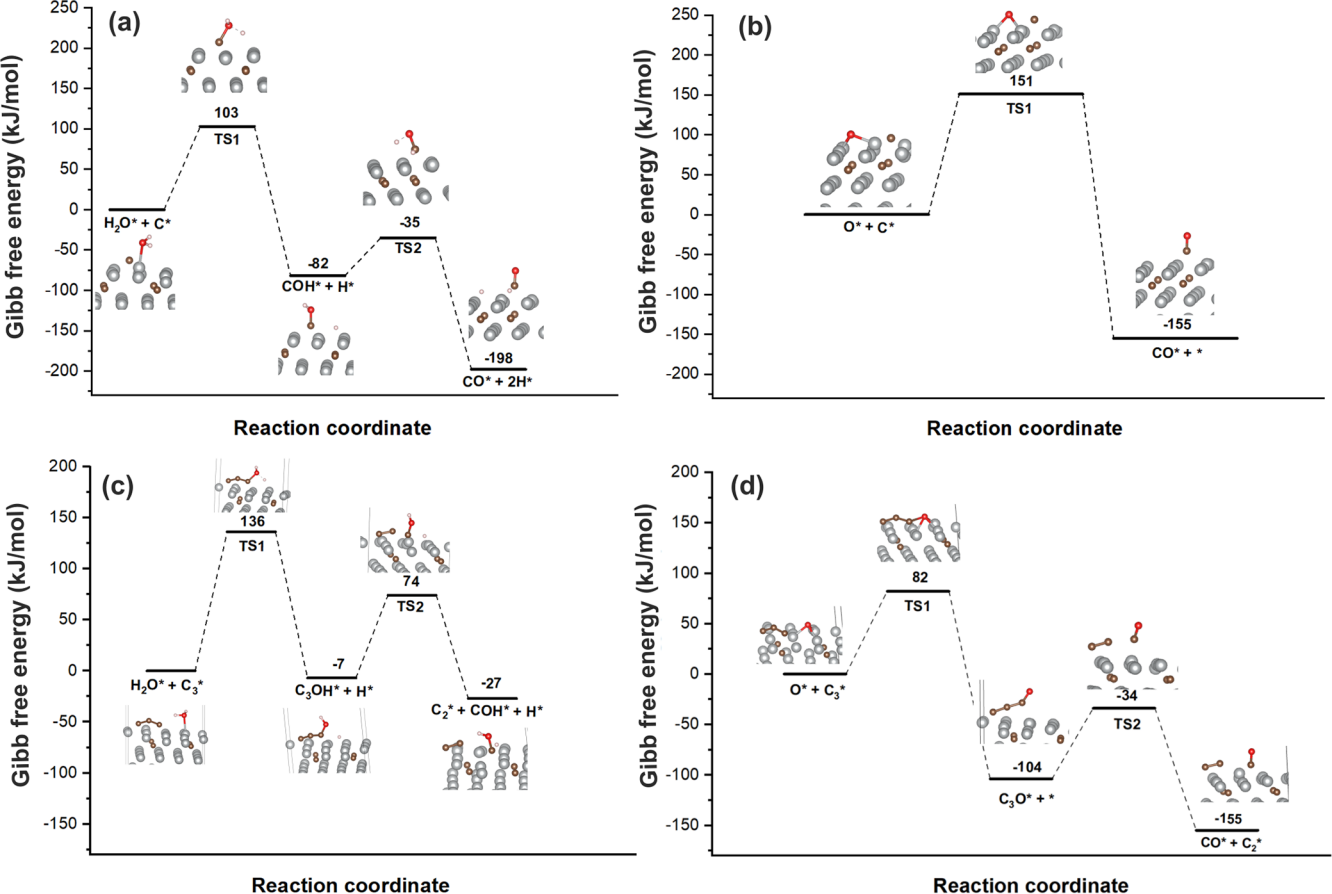
Reaction coordinates for H_2_O dissociation along with a carbon adatom at 1073K. H_2_O dissociation along with a carbon adatom: (a) carbon-assisted H_2_O dissociation pathway, (b) carbon oxidation by surface oxygen from H_2_O dissociation pathway. H_2_O dissociation along with a carbon trimer (C3): (c) carbon-assisted H_2_O dissociation pathway, (d) carbon oxidation by surface oxygen from H_2_O dissociation pathway.

Overall, for a carbon monomer, the reaction is most likely to occur via the carbon-assisted H_2_O dissociation pathway due to its lower activation barrier. However, if the carbon cluster is larger, such as in the case of a trimer, carbon-assisted H_2_O dissociation becomes less favorable due to a higher barrier. In such cases, H_2_O dissociation occurs first on the Ni surface to form atomic O, followed by a reaction with carbon to form CO. By either mechanism, the interaction between carbon and water can remove carbon from the Ni surface, thereby restoring active sites for CH_4_ decomposition, which contributes to enhanced CMD rates. In the presence of highly dispersed carbon monomers, the apparent barrier for reactions involving surface carbon and water is lower than that for methane activation. As a result, water interaction with surface species is expected to occur rapidly and approach quasi-equilibrium, rather than directly controlling the overall reaction rate. For larger carbon species, barriers are increased, and the impact of water may be diminished. Besides, we employ simplified carbon models (C adatom and a C_3_ trimer), to capture the basic trends associated with isolated carbon adsorption and the initial stages of carbon aggregation on Ni surfaces. In realistic catalytic environments, Ni surfaces may be populated by diverse carbon species, ranging from amorphous to graphitic structures, depending on carbon coverage and chemical potential. H_2_O interactions with these complex carbon phases may differ from those predicted by the idealized models used here; therefore, the present DFT results provide qualitative trends.

## 4 Conclusion

Water can play multiple roles during CMD, largely dependent on the timing of water introduction. When water is cofed with methane during initial stage of the reaction, it is hypothesized to interfere with the exsolution of Ni particles from nickel molybdate, which could reduce the availability of active sites and manifest as a decreased H_2_ production rate. After stabilizing the catalyst, water introduction enhances H_2_ production rates by facilitating more than one additional methane turnover per mol of H_2_O consumed (β > 2 mol H_2_/mol H_2_O), by gasifying carbon encapsulating Ni surfaces to expose more active sites. Water introduction enhances CMD rates over Ni–Mo/MgO for all tested water concentrations (8–24 Torr) and temperatures (725–800 °C). The decrease in apparent activation energy is attributed to an increase in the relative occurrence of methane turnovers performed on step-edge sites that facilitate lower activation energies. Further, DFT calculations indicate that the catalyst surface species significantly influence the mechanisms and energetics of water dissociation. The carbon-assisted H_2_O dissociation pathway is more likely for carbon monomers due to its lower activation barrier.

## Supplementary Material



## Notes

Reactor setup diagram, STEM images of catalysts, HRTEM images of CNTs, XRD and EDS/SEM results of CNTs, Calculation of CMD rates, *n*-Hexane reactions result, DFT calculations (PDF)
