# Supplementary material for: The Kinetic Consequences of Water on Catalytic Methane Pyrolysis
Source: ACS Catal. 2026 Feb 26;16(6):5907–19. doi: 10.1021/acscatal.6c00011 (PMC13010252; doi:10.1021/acscatal.6c00011)
Supplement: Supplementary file 1 [file cs6c00011_si_001.pdf]

The kinetic consequences of water on catalytic methane pyrolysis

Supporting Information

*Phuong T. Nguyen, Caleb Q. Bavlnka, Laura A. Gomez, Thy L.T. Ho, Ismaeel Alalq, Bin Wang, Daniel Resasco, Steven P. Crossley**

School of Sustainable Chemical, Biological and Material Engineering, University of Oklahoma, Norman, Oklahoma 73019, United States

* Email: stevencrossley@ou.edu

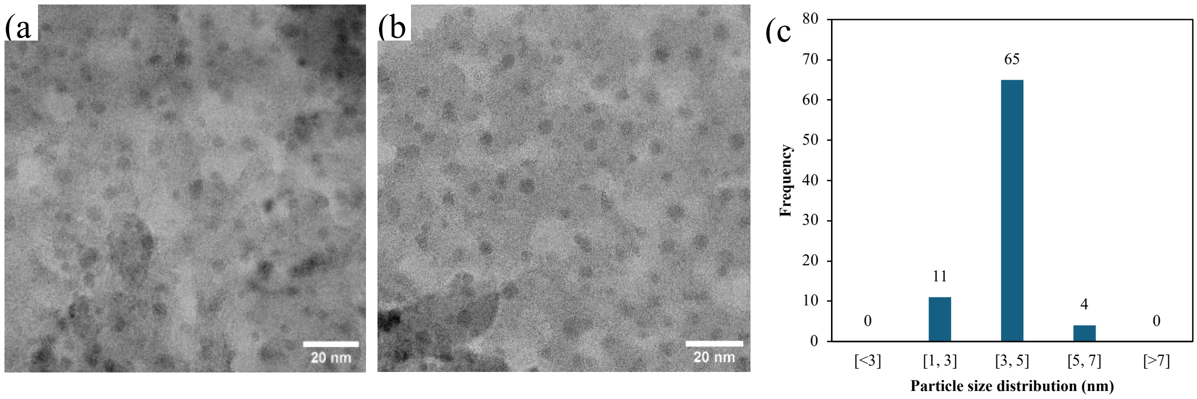

Figure S1. STEM images characterization of Ni particles (a-b) STEM results and (c) Ni particle size distribution for 5%Ni-25%Mo/MgO catalyst. The catalyst was pre-reduced under H_2 flow (100 mL/min) at 650 °C for 30 min at 1 atm.

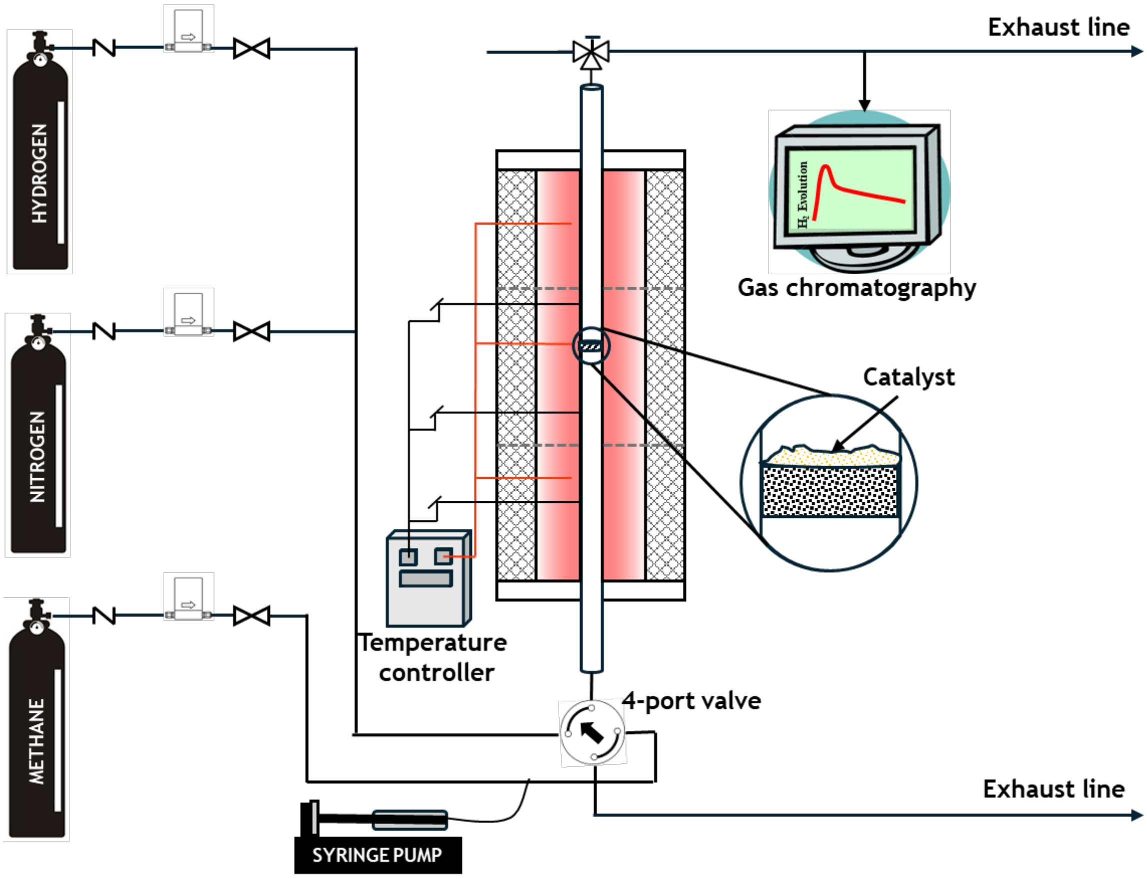

Figure S2. Reactor set-up diagram

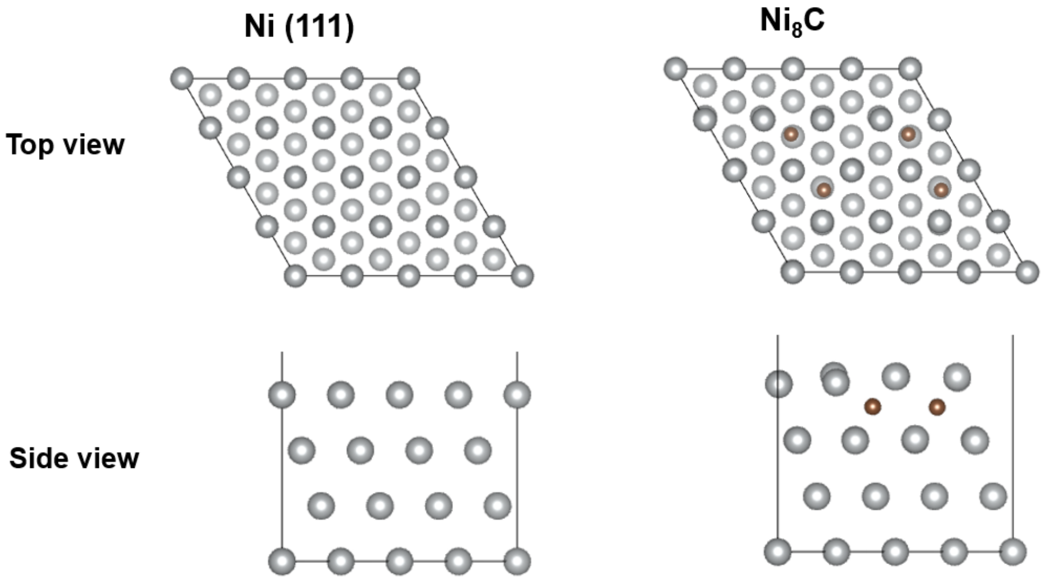

Figure S3. Top and side views of Ni (111)

Table S1. CNT yields after 3 hours as a function of the water partial pressure introduced at t = 0h. Reaction conditions: P_{Tot}= 1 atm, T_{rxn}= 800 ºC, W_{cat}= 100 mg, F_{CH4}= 100 mL/min, and t_{rxn} = 3h. All samples were previously reduced with H_2 at 650 ºC for 30 min.

Water Partial Pressure (Torr)	CNT Yield (g_{CNT}/g_{cat})	CNT Yield (g_{CNT}/g_{Ni})	Average TOF mol CH_4.mol Ni $^{-1}$.s^{-1}
0	10.56	211	0.096
2	10.44	209	0.095
5	9.80	196	0.089
8	8.94	179	0.081
16	7.76	155	0.071
24	7.72	154	0.070

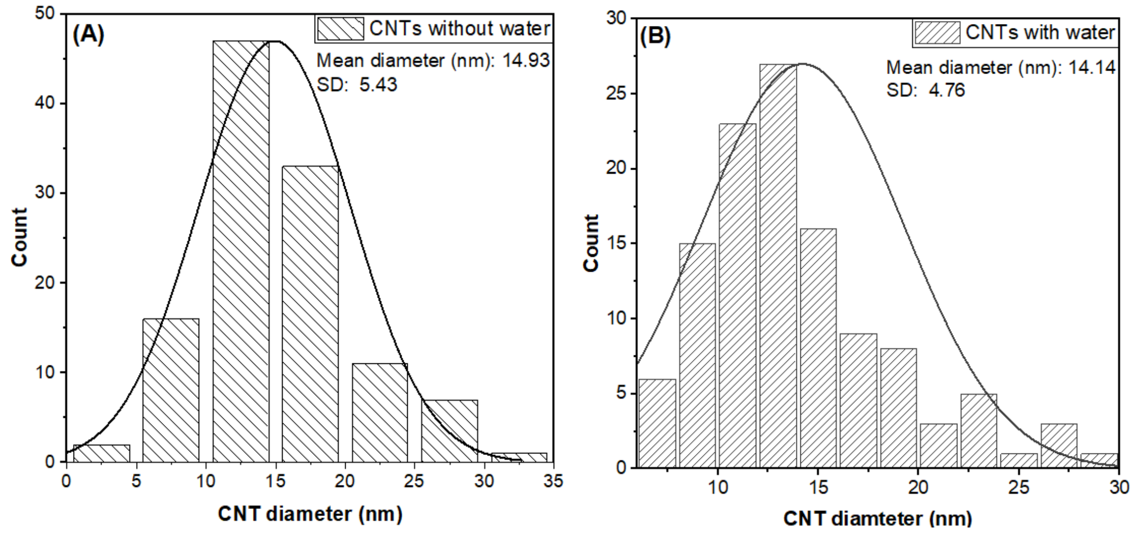

Figure S4. Distribution histograms and corresponding average CNT diameter size for: (A) CNTs without water and (B) with 16 Torr H_2O using 5%Ni-25%Mo/MgO catalyst for 3 hours. The materials synthesized for this analysis were prepared under the following conditions: P_{Total} = 1 atm, Flow of CH_4 = 100 mL/min, W_{cat} = 100 mg, and T_{rxn} = 800°C. The catalyst was pre-reduced under H_2 flow (200 mL/min) up to 650°C for 30 minutes.

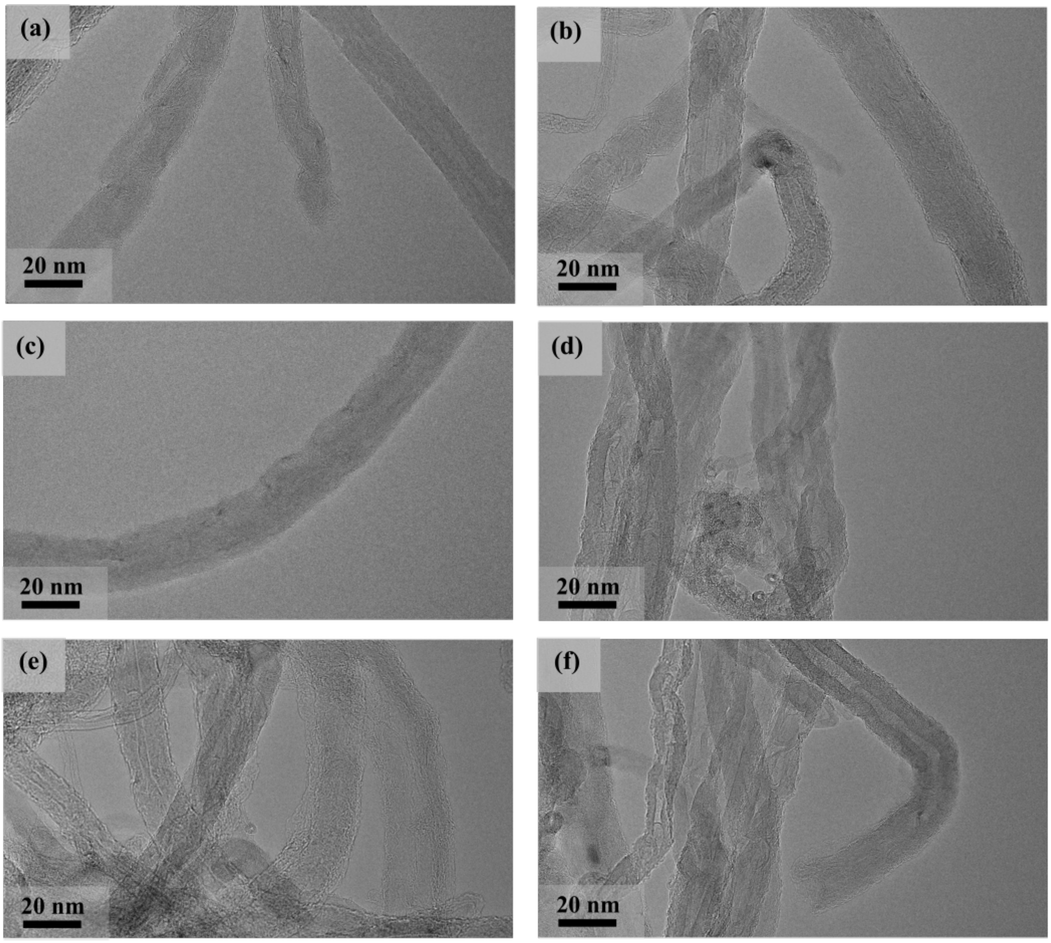

Figure S5. HRTEM images of carbon nanotubes grown on 5% Ni-25% Mo/MgO at 800 °C for 3 hours. Reaction conditions for reaction were: P_{Total}= 1 atm, Flow of CH_4= 100 mL/min, W_{cat}= 100 mg. The catalyst was previously reduced under H_2 flow (200 mL/min) up to 650 ºC for 30 min. The scale bar length for all TEM images is 20 nm.

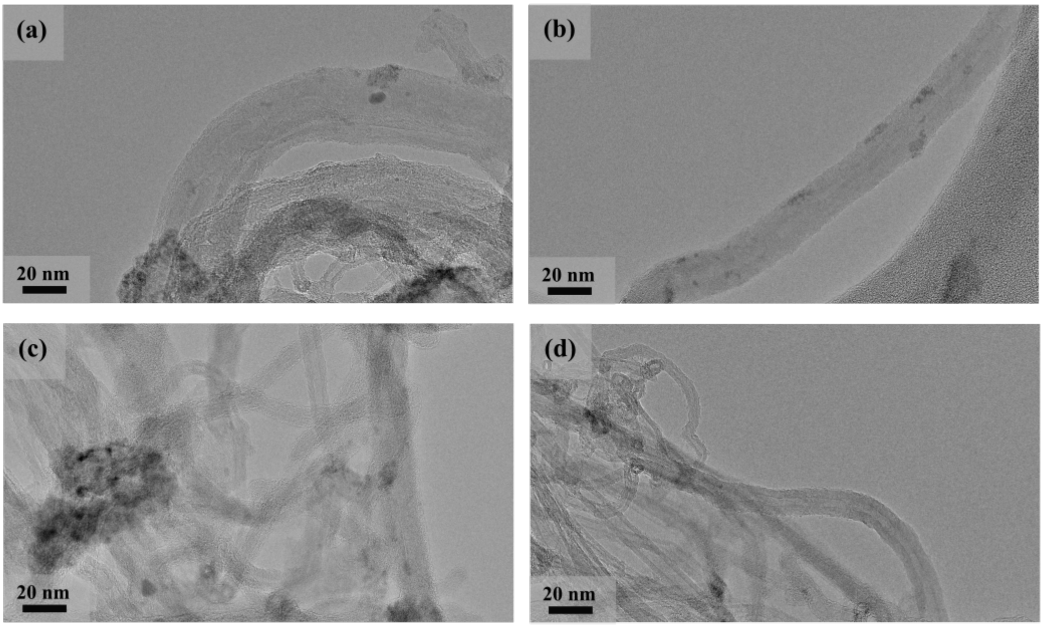

Figure S6. HRTEM images of carbon nanotubes grown on 5% Ni-25% Mo/MgO at 800 °C for 3 hours while co-feeding 16 Torr of water. Reaction conditions for reaction were: P_{Total}= 1 atm, Flow of CH_4= 100 mL/min, P_{H2O} = 16 Torr, W_{cat}= 100 mg. The catalyst was previously reduced under H_2 flow (200 mL/min) up to 650 ºC for 30 min. The scale bar length for all TEM images is 20 nm.

Table S2. Coefficients for fitted rate normalization polynomials of the form $Ax^3 + Bx^2 + Cx + D$ for CMD rates measured in the absence of water and after stabilization in the range of 725-800°C. For this function, x is equal to the nth data point collected, with each point collected ~7.5 minutes apart.

	**A**	**B**	**C**	**D**
**800°C**	-6.93E-06	1.01E-03	-5.15E-02	1.20E+00
**775°C**	-1.32E-05	2.03E-03	-1.03E-01	1.85E+00
**750°C**	-5.73E-06	8.55E-04	-4.22E-02	7.17E-01
**725°C**	-4.03E-06	6.39E-04	-3.33E-02	5.78E-01

Note S1. Definitions, calculations, and an example calculation of CMD rates

Gas-phase reactants and products were analyzed using an online gas chromatograph (GC). Methane (CH_4) was quantified using a flame ionization detector (FID), while hydrogen (H_2), and carbon monoxide (CO) were quantified using a thermal conductivity detector (TCD). GC detector responses were calibrated prior to reaction experiments using gas mixtures of known composition, and all gas-phase species were quantified as volume percentages (vol%). In the presence of water, hydrogen could be produced through secondary reactions (e.g., water-gas or water-gas shift reactions). To simplify the analysis and minimize uncertainty, methane consumption was used to calculate hydrogen production rates based on the stoichiometric relationship, since methane participates only in the primary decomposition reaction.

Methane conversion was calculated from the inlet and outlet methane molar flow rates according to:

$$X_{CH4} = \frac{F_{CH4,reacted}}{F_{CH4,in}}$$

Where $F_{CH4,reacted}$ and $F_{CH4,in}$ are the molar flow rate of reacted methane and the molar flow rate of inlet methane, respectively. $F_{CH4,in}$ was calculated using the ideal gas law based on the actual reactor operating pressure and temperature, and $F_{CH4,reacted}$ was determined by following stoichiometric relationships.

Catalytic methane decomposition (CMD) proceeds according to the overall stoichiometric reaction:

Main reaction:	$CH_{4(g)}$ →	$C_{(s)}$ +	$2H_{2(g)}$
Total inlet	*n*		
Reacted	*x*		*2x*
Outlet	*n-x*		*2x*
Total outlet	*n+x (mol.min^{-1})*		

$$[\%CH_4] = \frac{n-x}{n+x}$$

$$x = \frac{(1-[\%CH_4]).n}{(1+[\%CH_4])}\ (mol.min^{-1})$$

In the presence of water, the water gas reaction will occur:

	$C_{(s)}$ +	$H_2O_{(g)}$ →	$CO_{(g)}$ +	$H_{2(g)}$
Reacted	-	*z*	*z*	*z*
Outlet		*-z*	*z*	*2x+z*
Total outlet		*n+x+z (mol.min^{-1})*		

$$[\%CH_4] = \frac{n-x}{n+x+z}$$

$$x = \frac{n-[\%CH_4](n+z)}{(1+[\%CH_4])}\ (mol.min^{-1})$$

The resulting molar flow rates (mol. min^{-1}) were subsequently normalized by the catalyst mass to obtain reaction rates expressed in (mol. gcat^{-1}. h^{-1}) as r_{CH4}.

Hydrogen production rates are derived from the reacted methane rate above, following the stoichiometric relationship:

$$r_{\mathrm{H_2}} = 2 \times r_{\mathrm{CH_4}}$$

The molar flow rate of CO was calculated as $F_{\mathrm{CO}} = [\%\mathrm{CO}] \times F_{\mathrm{total,in}}$, where $[\%\mathrm{CO}]$ is the measured CO volume fraction and $F_{\mathrm{total,in}}$ is the total inlet molar flow rate. The amount of water consumed was subsequently determined from the CO molar flow rate based on the corresponding stoichiometric relationships.

Calculations involving the water-gas shift reaction were performed assuming equilibrium, and elemental balances of O, C, and H were used to validate the results.

$$CO_{(g)} + H_2O_{(g)} \leftrightarrow CO_{2(g)} + H_{2(g)}$$

$$K_{eq} = \frac{[CO_2][H_2]}{[CO][H_2O]}$$

Calculation methods for measured, baseline, and corrected CMD rate values

The ***measured*** H_2 production rate is the rate value that is obtained directly from experimental results, which consists of a convolution of methane conversion and deactivation effects.

$$r_{measured} = f(r_{CH4}, \varphi(t))$$

The ***baseline*** CMD rate is the rate value that is identified at 3 hours TOS, as the reaction approaches pseudo steady state, just before water introduction. This rate represents the CMD rate after catalyst stabilization but in the absence of further time-dependent catalyst deactivation and will be the reference for calculated rate corrections after t = 3 hours.

$$r_{baseline} = f(r_{CH4})$$

$$r_{baseline} = r_{measured}|_{t=3\ hrs}$$

The ***corrected*** CMD rate is a calculated rate value that is produced from the fitting of a cubic polynomial from Table S2 to CMD rates measured in the absence of water. This polynomial represents the CMD rate at a given time with corrections for time-dependent deactivation, and the value of this function varies with time. Thus, the corrected rate is the rate of CMD as though further deactivation does not occur with respect to time.

$$r_{corrected} = f(r_{CH4})$$

$$r_{corrected} = r_{measured} + \left(r_{baseline} - f_{polynomial}(t)\right)$$

An example calculation for a single point collected in the scenario represented in Figure 3, which occurred at t = 5 hrs, 800°C, and 16 Torr H_2O, is presented here:

$$r_{measured}|_{t=5\ hrs} = 0.423 \frac{mol\ H_2}{g_{cat} * hr}$$

$$r_{baseline} = r_{measured}|_{t=3\ hrs} = 0.417\ \frac{mol\ H_2}{g_{cat} * hr}$$

$$f_{polynomial|_{t=5\ hrs}} = 0.305 \frac{mol\ H_2}{g_{cat} * hr}$$

$$r_{corrected|_{t=5\ hrs}} = r_{measured|_{t=5\ hrs}} + \left(r_{baseline} - f_{polynomial|_{t=5\ hrs}}\right)$$

$$r_{corrected|_{t=5\ hrs}} = 0.423 + (0.417 - 0.305) = 0.535\ \frac{mol\ H_2}{g_{cat} * hr}$$

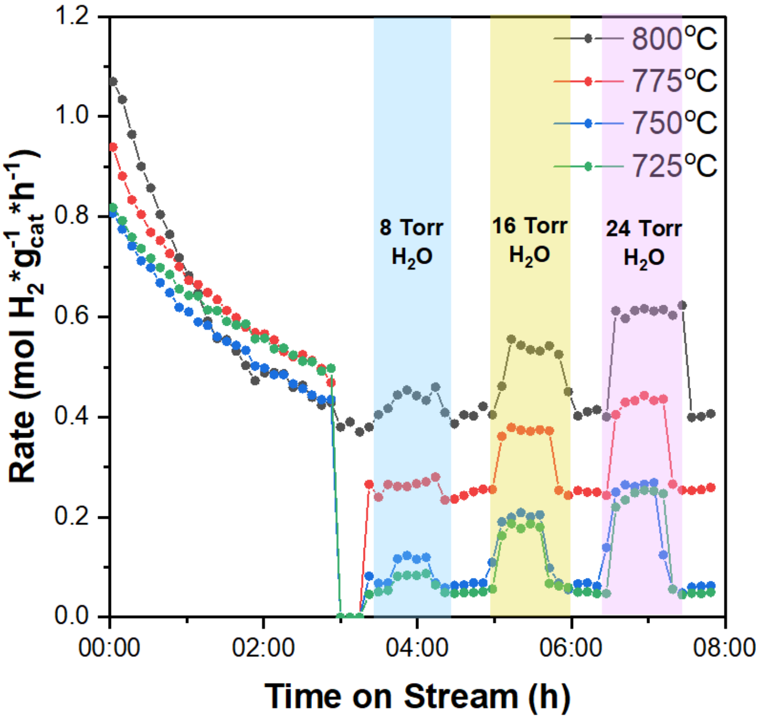

Figure S7. Corrected H_2 hydrogen production rates as a function of both water partial pressure and temperature after catalyst stabilization. Reaction conditions for all reactions were: P_{Tot}= 1 atm, Flow of CH_4= 100 mL/min, $W_{cat.}$= 100 mg, and T_{rxn}= 725-800ºC. The catalyst was previously reduced under H_2 flow (200 mL/min) at 650ºC for 30 min. Water partial pressure was varied from 0-24 Torr. The decrease in rate at 3 hours resulted from changing the feed to nitrogen while changing temperature; the following increase in rate resulted from methane introduction at the designated temperature.

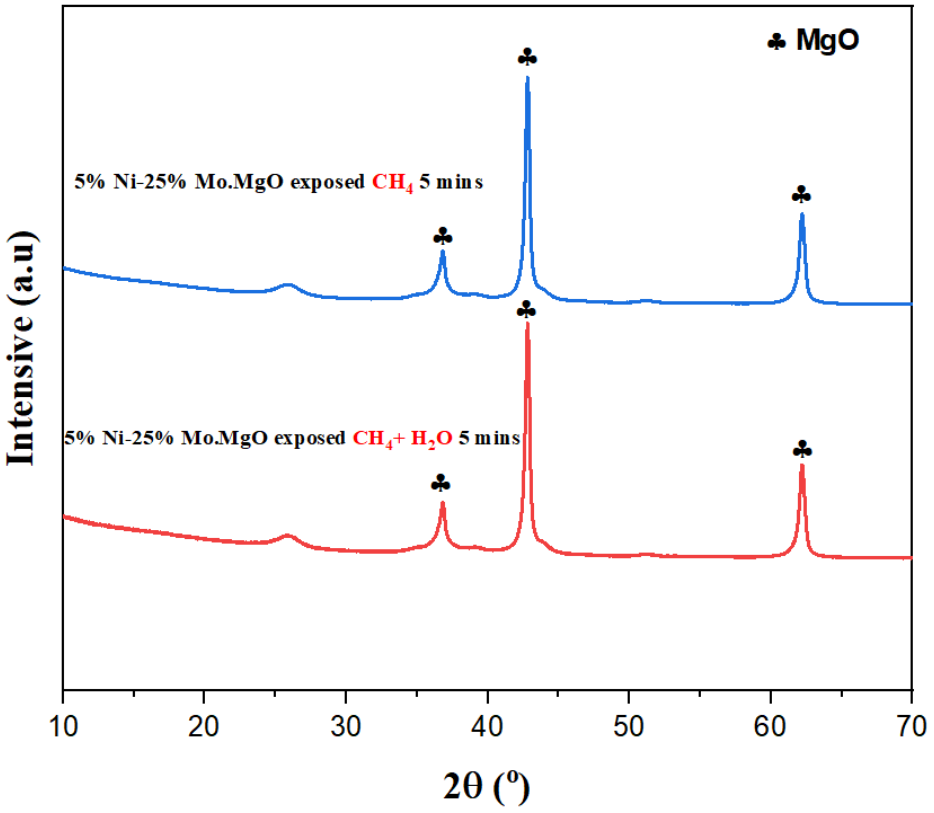

Figure S8. XRD diagrams of 5% Ni-25% Mo/MgO after 5 mins exposure to methane and methane with water.

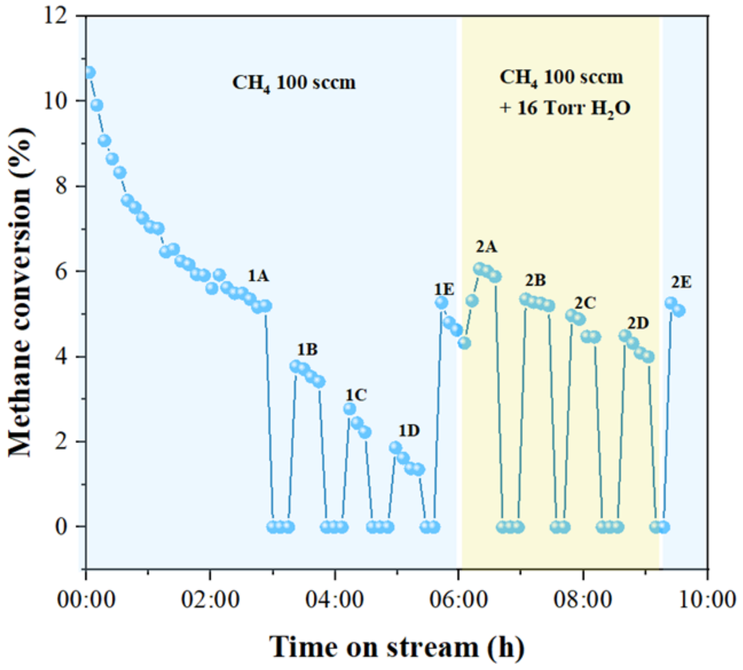

A	B	C	D	E
825 °C	800 °C	775 °C	750 °C	825 °C

Figure S9. Methane conversion as a function of time with correction for time-dependent deactivation effects. Reaction conditions for all reactions were: P_{Total}= 1 atm, Flow of CH_4= 100 mL/min, $W_{cat.}$= 100 mg, and $T_{reaction}$= 750-825ºC. The catalyst was previously reduced under H_2 flow (200 mL/min) at 650 ºC for 30 mins. Water pressure is 0 Torr until water was introduced at 16 Torr at t = 6:15.

Table S3. Comparison of the activation energy of Ni-based catalysts reported in the literature for methane decomposition. DFT-calculated metal catalyst activities are denoted by [*]

Catalyst	Ea (kJ/mol)	Temp. (ºC)	Pressure	Conversion (%)	Gas Flow	Reference
Without catalyst	312-450	N/A	N/A	N/A	-	[1]
Ni/MgO	87.8	725-800	5.0 kPa CH_4	N/A	CH_4/N_2	[2]
Mo/Ni/MgO	200.3	900-1200	5.0 kPa CH_4	N/A	CH_4/N_2	[2]
13% Ni/TiO_2	60	550-900	1 atm	N/A	CH_4/Ar = 1:1	[3]
Ni-Cu/MgO	50.4	550-650	1 atm	N/A	$CH_4/H_2/N_2$ CH_4 = (6-10%) H_2 = (5-8%)	[4]
Ni-Cu/SiO_2	90	450-590	N/A	N/A	CH_4+ H_2	[5]
n-Ni/SiO_2	61.1	550-650	0.2-0.8 atm	3-8%	CH_4+ N_2	[6]
*Ni (111)	105	N/A	N/A	N/A	-	[7]
*Ni (211)	90	N/A	N/A	N/A	-	[7]

Table S4. Comparison of the average TOF estimated from data reported in the literature on metal-based catalysts for the CMD reaction. (a) This indicates data from our own catalysts reported in this paper.

**Average TOF mol CH_4.mol metal $^{-1}$.s^{-1}**	**Catalyst**	**Temp. (°C)**	**Partial pressure CH_4**	**Flow rate**	**Time run**	**Ref**
~0.012	10%Ni-1%Cu/CNT	600 °C	0.3	CH_4:N_2 = 3:7, F_{Total} = 30 ml/min	20 mins	[8]
0.109	5%Fe-20%Mo/MgO	800 °C	1	0.0064 mol/s CH_4	60 mins	[2]
0.068	5%Ni-30%Mo/MgO	800 °C	1	0.0064 mol/s CH_4	60 mins	[2]
0.087	5%Co-20%Mo/MgO	800 °C	1	0.0064 mol/s CH_4	60 mins	[2]
0.069	Fe-1Mo/MgO (0.03 mol Fe/mol MgO 1 mol Mo/mol Fe)	850 °C	1	20 ml/min CH_4	30 mins	[9]
0.12	5%Ni-20%Mo/MgO	800 °C	1	100 ml/min CH_4	30 mins	*(a)*
0.11	5%Ni-20%Mo/MgO	800 °C	1	100 ml/min CH_4	60 mins	*(a)*
0.096	5%Ni-20%Mo/MgO	800 °C	1	100 ml/min CH_4	180 mins	*(a)*

Note S2. **Estimate internal diffusion criteria**.

The potential influence of internal diffusion was evaluated using the Weisz–Prater criterion.

$$C_{WP} = \frac{\rho_{cat} r_{obs} R_p^2}{C_{CH4} D_{eff}}$$

where ρ_{cat} is the apparent catalyst density (kg·m^{-3}), r_{obs} is the observed CMD rate normalized per unit catalyst mass ($mol\ CH_4 . kg^{-1} s^{-1}$), R_p is the catalyst particle radius (m), C_{CH_4} is the

methane concentration at the particle surface (mol·m^{-3}), and D_{eff} is the effective diffusivity of methane within the catalyst particle (m^2·s^{-1}).

For the 5%Ni-25%Mo/MgO catalyst, the catalyst density of $\rho_{\text{cat}} = 4000$ kg·m^{-3} was used. The observed CMD rate was taken from steady-state experimental data at 800 °C and expressed on a mass basis as $r_{\text{obs}} = 3.375\ (mol\ CH_4 . kg^{-1} s^{-1})$. The catalyst particle radius was estimated as $R_p = 125$ µm.

The methane concentration at the particle surface was calculated using the ideal gas law. Using atm, $R = 0.08215 \times 10^{-3}$(m³·atm·mol⁻¹·K⁻¹), and $T = 1073$K, $C_{CH_4}$was estimated to be:

$$C_{CH4} = \frac{P_{CH4}}{RT} = \frac{1\ (atm)}{0.08215 \times 10^{-3} \left(\frac{m^3 * atm}{mol * K}\right) \times 1073\ (K)} \approx 11.34\ (mol * m^{-3})$$

The effective diffusivity of methane within the porous catalyst was estimated as:

$$D_{eff} = \frac{\varepsilon_p}{\tau} D_{CH_4}$$

Where ε_p is particle porosity and τ is the tortuosity, D_{CH_4} is he gas-phase diffusion coefficient of methane. In the absence of direct measurements of particle porosity and tortuosity for the present Ni-Mo/MgO catalyst, these parameters were estimated based on typical values reported for porous oxide supports. Since the MgO support constitutes the majority of the catalyst volume and therefore governs the pore network relevant for internal diffusion, particle porosity and tortuosity were approximated using representative ranges commonly adopted for porous oxide supports (ε_p = 0.3–0.5, τ = 3–5). These values are widely used as reasonable estimates in the absence of direct pore network measurements. The gas-phase diffusion of methane at 1 atm can be estimated using D ~ $T^{1.5}$/P (where T is Kelvin). Using $D_{25°C}$ = 0.22 cm^2·s^{-1}, the diffusion coefficient at 800 °C was estimated as:

$$D_{800} \approx D_{25} \left(\frac{1073K}{298K}\right)^{1.5} \approx 0.22 \frac{cm^2}{s} \times (3.60)^{1.5} \approx 1.5\ \frac{cm^2}{s}$$

Using $\varepsilon_p = 0.4, \tau = 4$: $D_{eff} = 1.5 \times 10^{-4}\ m^2 * s^{-1}$

The Weisz–Prater criterion can be calculated as:

$$C_{WP} = \frac{\rho_{cat} r_{obs} R_p^2}{C_{CH4} D_{eff}}$$

$$= \frac{4000\ (kg * m^{-3}) \times 3.375\ (mol\ CH_4 * kg^{-1} s^{-1}) \times (125 * 10^{-6})^2 (m^2)}{11.34\ (mol * m^{-3}) \times (1.5 * 10^{-4})(m^2 * s^{-1})}$$

$$= 0.124$$

Since $C_{WP} < 0.3$, the internal diffusion limitations are negligible under the investigated conditions, confirming that the reported CMD rates reflect intrinsic catalytic kinetics.

Using a conservative Weisz–Prater threshold of $C_{WP} = 0.3$, the maximum allowable observed rate before the onset of internal diffusion limitations was estimated to be approximately 8.16 $mol\ CH_4 * kg^{-1} * s^{-1}$ for catalyst particles with a radius of 125 µm.

$$r_{obs,max} = \frac{C_{CH4} D_{eff} C_{WP}}{R_p^2 \rho_{cat}} = \frac{11.34\ (mol * m^{-3}) \times (1.5 * 10^{-4})(m^2 * s^{-1}) \times 0.3}{(125 * 10^{-6})^2 (m^2) \times\ 4000\ (kg * m^{-3})}$$

$$= 8.16\ mol\ CH_4 * kg^{-1} * s^{-1}$$

All experimentally measured CMD rates fall well below this value, confirming that the reported kinetics are not influenced by internal diffusion.

Note S3:

Surface probes of the reactive surface with n-hexane at 300°C reveal diminished dehydrogenation and aromatization rates upon water introduction, even at lower temperatures. The only reaction products observed were dehydrogenated n-hexane and aromatics. No isomerization or cracking products were observed. Continuous water introduction alongside the reactant led to reversible deactivation. Future studies will expand upon these results in an effort to quantify Mo_2C surface disruptions under reaction conditions.

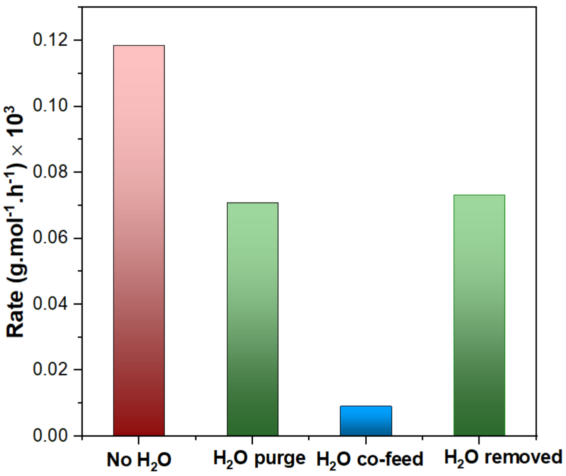

Figure S10. Reaction rate of n-hexane at 300°C over a 5% Ni-25% Mo/MgO catalyst after 5 min exposure to CH_4 at 800°C, followed by cooling and introduction to water and n-hexane as described in the experimental procedures.

Note S4. Mechanistic insights into water-assisted CMD on bare Ni (111) and Ni_8C

Under the experimental conditions, where the carbon chemical potential within the Ni bulk is lower than on its surface, the formation of carbides is anticipated[10]. Several configurations were investigated, including a bare Ni surface and a Ni carbide surface; the latter was modelled as Ni (111) with four carbon atoms incorporated into the first lattice layer (Ni_8C), see **Figure S3**. The dissociation pathways of H_2O into surface OH, H, and O were examined on these two surfaces. On the Ni(111) surface, the most stable adsorption sites for OH, H, and O species are found at the FCC sites [11]. For H_2O adsorption, the most stable configuration is located at the top site, where the H_2O molecule aligns nearly parallel to the nickel surface [11]. As shown in **Figure S11**, the dissociation of H_2O at the top site into OH and H shows an activation barrier of 75 kJ/mol, with a reaction energy of -39 kJ/mol. Subsequently, the dissociation of OH into H and O exhibits an energy barrier of 80 kJ/mol, consistent with previous studies, and the reaction energy is exothermic at -37 kJ/mol. [12] In contrast, for the Ni_8C surface, the activation energies for the dissociation of H_2O into OH and H, as well as the subsequent dissociation of OH into H and O, are 108 kJ/mol and 95 kJ/mol, respectively. Both values are higher than those

observed for the pure Ni (111) surface. These differences may be attributed to the carbide layer on the dissociation process of H_2O, destabilizing the transition state structure and leading to increased activation barriers. Overall, under the experimental conditions, it is likely that H_2O dissociates readily into H and O species on both catalyst surfaces.

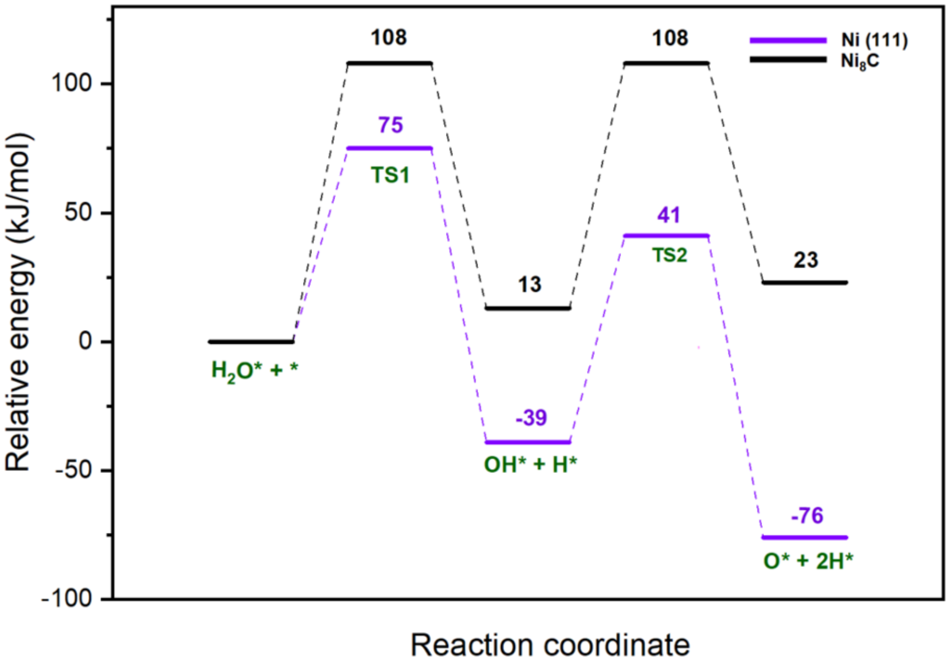

Figure S11. Reaction coordinate for H_2O dissociation on bare Ni (111) and Ni_8C

Proposed elementary steps for H_2O dissociation with a carbon adatom and with a carbon trimer.

a) H_2O dissociation with a carbon adatom

Pathway (a): Carbon-assisted H_2O dissociation

$H_2O^* + C^* \rightarrow COH^* + H^*$

$COH^* + H^* \rightarrow CO^* + 2H^*$

Pathway (b): Carbon oxidation by surface oxygen from H_2O dissociation

$H_2O^* \rightarrow OH^* + H^*$

$OH^* \rightarrow O^* + H^*$

$O^* + C^* \rightarrow CO^*$

b) H_2O dissociation with a carbon trimer (C_3)

Pathway (c): Carbon-assisted H_2O dissociation

$H_2O^* + C_3^* \rightarrow C_3OH^* + H^*$

$C_3OH^* + * \rightarrow C_2^* + COH^*$

Pathway (d): Carbon oxidation by surface oxygen from H_2O dissociation

$H_2O^* + * \rightarrow OH^* + H^*$

$OH^* + * \rightarrow O^* + H^*$

$O^* + C_3^* \rightarrow C_3O^{**}$

$C_3O^{**} \rightarrow CO^* + C_2^*$

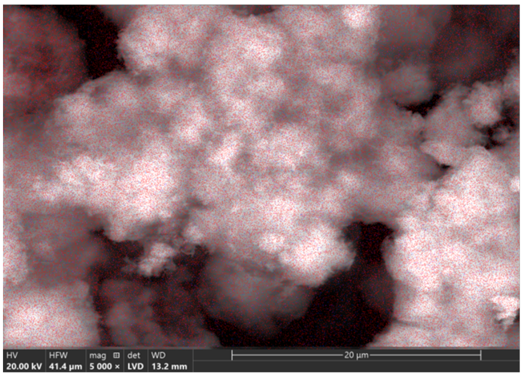

Element	Atomic %	Atomic % Error	Weight %	Weight % Error
O	58.9	0.2	40.9	0.1
Mg	35.3	0.1	37.2	0.1
Ni	1.2	0.0	3.1	0.0
Mo	4.5	0.0	18.8	0.0

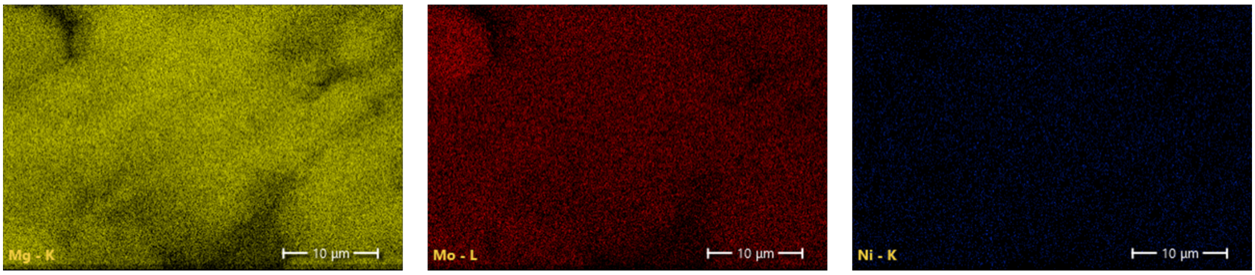

Figure S12. SEM-EDS of the fresh catalyst 5%Ni-25%Mo/MgO.

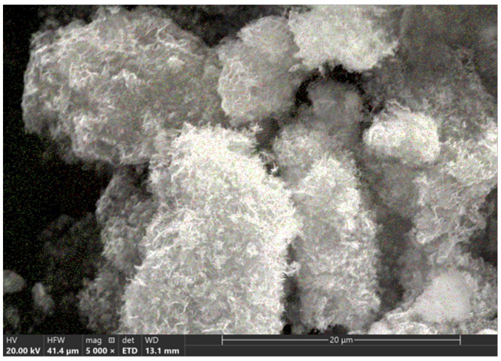

Element	Atomic %	Atomic % Error	Weight %	Weight % Error
C	39.3	0.5	25.3	0.3
O	35.3	0.3	30.3	0.3
Mg	22.0	0.1	28.7	0.1
Ni	0.8	0.0	2.5	0.1
Mo	2.6	0.0	13.2	0.1

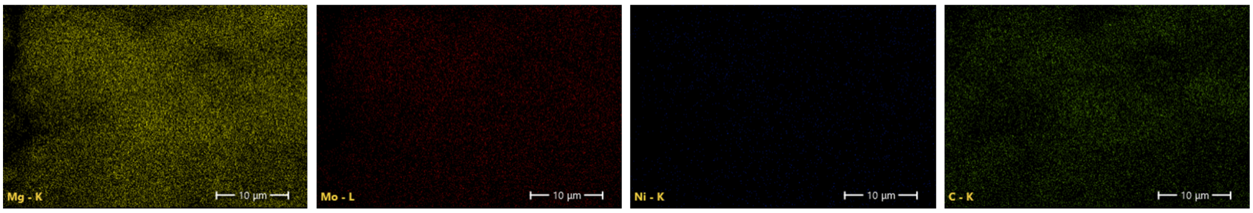

Figure S13. SEM-EDS for 5%Ni-25%Mo/MgO after 5 minutes exposure to CH_4. Reaction conditions: P_{Tot}= 1 atm, T_{rxn}= 800 °C, W_{cat}= 100 mg, F_{CH4}= 100 mL/min, and t_{rxn} = 5 mins. The catalyst was previously reduced with H_2 at 650 °C for 30 min.

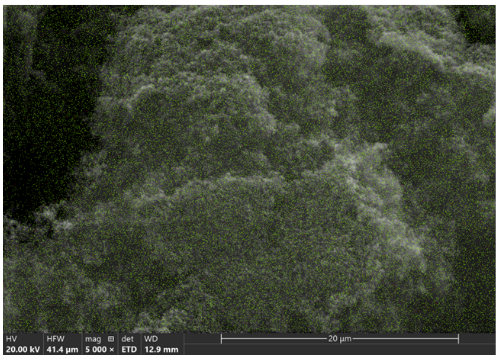

Element	Atomic %	Atomic % Error	Weight %	Weight % Error
C	89.4	0.3	82.8	0.3
O	7.3	0.2	9.1	0.3
Mg	2.9	0.0	5.5	0.0
Ni	0.1	0.0	0.4	0.0
Mo	0.3	0.0	2.2	0.0

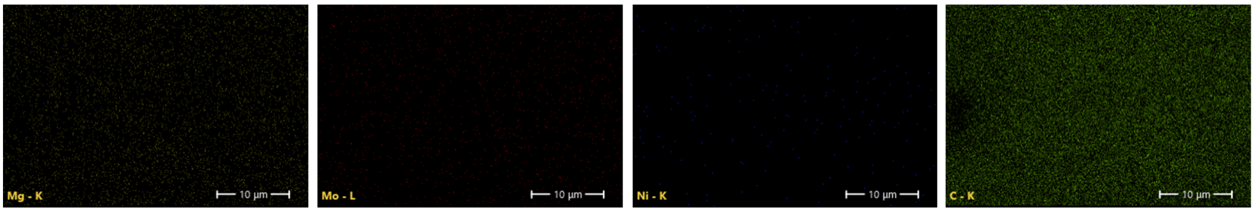

Figure S14. SEM-EDS for 5%Ni-25%Mo/MgO after 3 hours CNT growth. Reaction conditions: P_{Tot}= 1 atm, T_{rxn}= 800 °C, W_{cat}= 100 mg, F_{CH4}= 100 mL/min, and t_{rxn} = 3h. The catalyst was previously reduced with H_2 at 650 °C for 30 min.

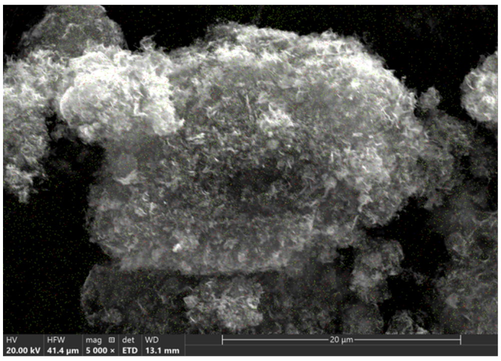

Element	Atomic %	Atomic % Error	Weight %	Weight % Error
C	50.4	0.5	35.6	0.3
O	29.9	0.3	28.1	0.3
Mg	17.5	0.1	25.0	0.1
Ni	0.6	0.0	2.0	0.0
Mo	1.6	0.0	9.3	0.1

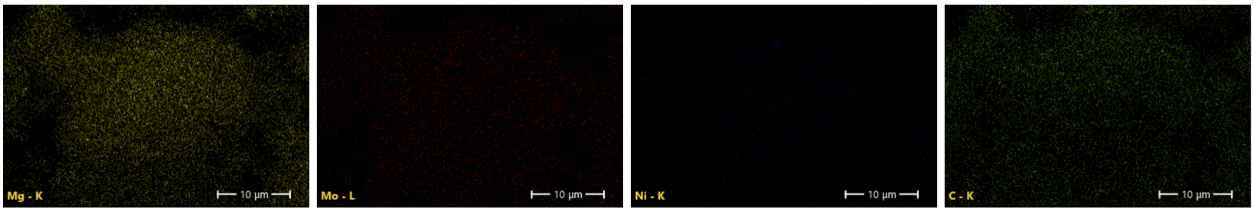

Figure S15. SEM-EDS for 5%Ni-25%Mo/MgO after 5 minutes exposure to CH_4 + H_2O. Reaction conditions: P_{Tot}= 1 atm, T_{rxn}= 800 °C, W_{cat}= 100 mg, F_{CH4}= 100 mL/min, P_{H2O} = 16 Torr and t_{rxn} = 5 mins. The catalyst was previously reduced with H_2 at 650 °C for 30 min.

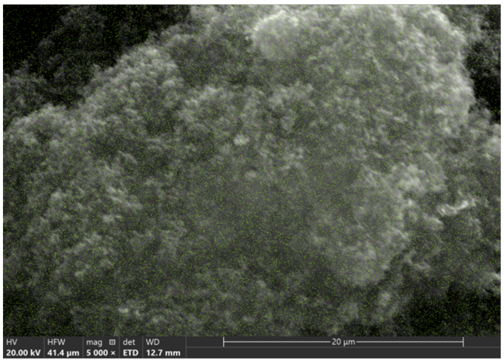

Element	Atomic %	Atomic % Error	Weight %	Weight % Error
C	81.9	0.3	74.1	0.3
O	14.5	0.3	17.4	0.3
Mg	3.3	0.0	6.0	0.0
Ni	0.1	0.0	0.6	0.0
Mo	0.3	0.0	2.0	0.0

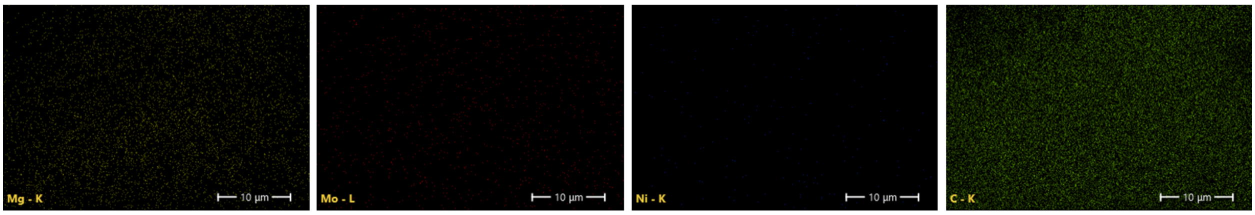

Figure S16. SEM-EDS for 5%Ni-25%Mo/MgO after 3 hours CNT growth with water. Reaction conditions: P_{Tot}= 1 atm, T_{rxn}= 800 °C, W_{cat}= 100 mg, F_{CH4}= 100 mL/min, P_{H2O} = 16 Torr and t_{rxn} = 3h. The catalyst was previously reduced with H_2 at 650 °C for 30 min.

REFERENCES

(1) Toncu, D.-C.; Toncu, G.; Soleimani, S. On methane pyrolysis special applications. In *IOP Conference Series: Materials Science and Engineering*, 2015; IOP Publishing: Vol. 95, p 012026. https://doi.org/10.1088/1757-899X/95/1/012026.

(2) Ni, L.; Kuroda, K.; Zhou, L.-P.; Ohta, K.; Matsuishi, K.; Nakamura, J. Decomposition of metal carbides as an elementary step of carbon nanotube synthesis. *Carbon* **2009**, *47* (13), 3054-3062.

(3) Sharif Zein, S. H.; Mohamed, A. R.; Talpa Sai, P. S. Kinetic studies on catalytic decomposition of methane to hydrogen and carbon over Ni/TiO2 catalyst. *Industrial & engineering chemistry research* **2004**, *43* (16), 4864-4870. DOI: https://doi.org/10.1021/ie034208f.

(4) Borghei, M.; Karimzadeh, R.; Rashidi, A.; Izadi, N. Kinetics of methane decomposition to COx-free hydrogen and carbon nanofiber over Ni–Cu/MgO catalyst. *international journal of hydrogen energy* **2010**, *35* (17), 9479-9488. DOI: https://doi.org/10.1016/j.ijhydene.2010.05.072.

(5) Alstrup, I.; Tavares, M. T. Kinetics of carbon formation from CH4+ H2 on silica-supported nickel and Ni-Cu catalysts. *Journal of Catalysis* **1993**, *139* (2), 513-524. DOI: https://doi.org/10.1006/jcat.1993.1045.

(6) Ashik, U.; Daud, W. W.; Abbas, H. F. Methane decomposition kinetics and reaction rate over Ni/SiO2 nanocatalyst produced through co-precipitation cum modified Stöber method. *International Journal of Hydrogen Energy* **2017**, *42* (2), 938-952.

(7) Abild-Pedersen, F.; Lytken, O.; Engbæk, J.; Nielsen, G.; Chorkendorff, I.; Nørskov, J. K. Methane activation on Ni (1 1 1): Effects of poisons and step defects. *Surface Science* **2005**, *590* (2-3), 127-137. https://doi.org/10.1016/j.susc.2005.05.057.

(8) Lopez-Ruiz, J. A.; Jiang, Y.; Riedel, N. W.; Weber, R. S.; Jiang, C.; Xu, M.; Hu, J.; Dagle, R. A. CO2-free (turquoise) H2 at $1/kg via thermocatalytic decomposition of methane and sale of solid carbon co-product. An experimental and techno-economic analysis. *International Journal of Hydrogen Energy* **2026**, *207*, 153492.

(9) Pan, Z.; Krumeich, F.; Ziemiański, P. P.; van Bokhoven, J. A. Tunable synthesis of carbon nanotubes via methane catalytic pyrolysis by adjusting Mo incorporation in Fe/MgO. *Physical Chemistry Chemical Physics* **2025**, *27* (35), 18302-18308.

(10) Yang, Z.; Wang, Q.; Shan, X.; Yang, S.-W.; Zhu, H. Theoretical investigation on carbon nucleation on nickel carbides at initial stages of single-walled carbon nanotube formation. *Physical Chemistry Chemical Physics* **2014**, *16* (36), 19654-19660. DOI: https://doi.org/10.1039/C4CP02837F.

(11) Hou, X.; Qi, L.; Li, W.; Zhao, J.; Liu, S. Theoretical study on water adsorption and dissociation on the nickel surfaces. *Journal of Molecular Modeling* **2021**, *27* (2), 36. https://doi.org/10.1007/s00894-020-04662-4.

(12) Pozzo, M.; Carlini, G.; Rosei, R.; Alfè, D. Comparative study of water dissociation on Rh(111) and Ni(111) studied with first principles calculations. *The Journal of chemical physics* **2007**, *126* (16), 164706-164706. From Unbound Medicine PRIME.
